# Dissecting the Interactions between Chlorin e6 and Human Serum Albumin

**DOI:** 10.3390/molecules28052348

**Published:** 2023-03-03

**Authors:** Alessia Marconi, Edoardo Jun Mattioli, Filippo Ingargiola, Giulia Giugliano, Tainah Dorina Marforio, Luca Prodi, Matteo Di Giosia, Matteo Calvaresi

**Affiliations:** Dipartimento di Chimica “Giacomo Ciamician”, Alma Mater Studiorum—Università di Bologna, Via Francesco Selmi 2, 40126 Bologna, Italy

**Keywords:** chlorin e6, human serum albumin (HSA), docking, MD simulations, reactive oxygen species (ROS), photosensitizer (PS)

## Abstract

Chlorin e6 (Ce6) is among the most used sensitizers in photodynamic (PDT) and sonodynamic (SDT) therapy; its low solubility in water, however, hampers its clinical exploitation. Ce6 has a strong tendency to aggregate in physiological environments, reducing its performance as a photo/sono-sensitizer, as well as yielding poor pharmacokinetic and pharmacodynamic properties. The interaction of Ce6 with human serum albumin (HSA) (i) governs its biodistribution and (ii) can be used to improve its water solubility by encapsulation. Here, using ensemble docking and microsecond molecular dynamics simulations, we identified the two Ce6 binding pockets in HSA, i.e., the Sudlow I site and the heme binding pocket, providing an atomistic description of the binding. Comparing the photophysical and photosensitizing properties of Ce6@HSA with respect to the same properties regarding the free Ce6, it was observed that (i) a red-shift occurred in both the absorption and emission spectra, (ii) a maintaining of the fluorescence quantum yield and an increase of the excited state lifetime was detected, and (iii) a switch from the type II to the type I mechanism in a reactive oxygen species (ROS) production, upon irradiation, took place.

## 1. Introduction

**Scheme 1:**
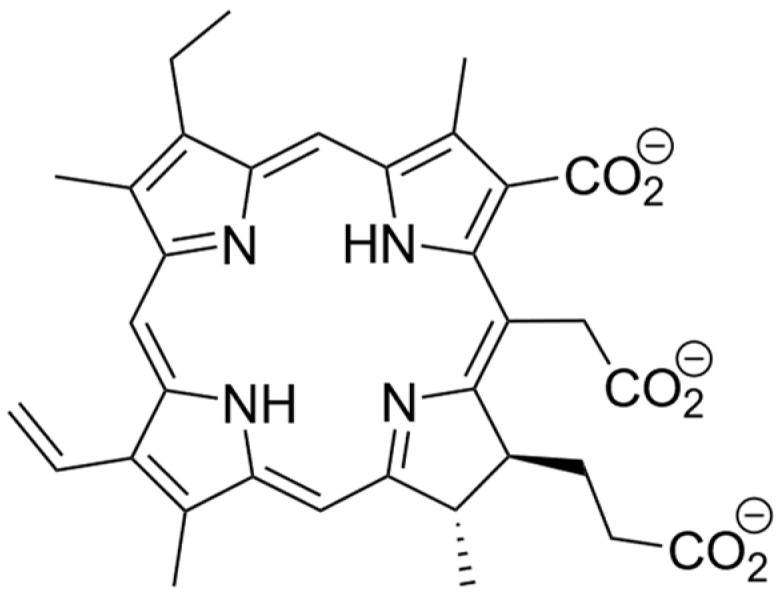
Molecular structure of Ce6.

Chlorin e6 (Ce6) and its derivatives are among the most important photosensitizers (PSs) used in photodynamic therapy (PDT) [1,2]. Ce6 (Scheme 1) is characterized by the following: (i) a strong absorption in the red region of the visible spectra (around 660 nm, in the first phototherapeutic window) [2]; (ii) an excellent photoconversion efficiency (at a physiological pH, the quantum yield of singlet oxygen is 0.64 [3]); (iii) an intense fluorescence that may be used for imaging (i.e., in theranostic applications) [2]; and (iv) the possibility to activate the production of a reactive oxygen species (ROS) using ultrasounds (sonosensitization) [2,4,5,6] as an alternative to light (photosensitization).

However, like many other hydrophobic photosensitizers, the solubility of Ce6 in water is low, thus arising in the need for an appropriate formulation [2,7] for its clinical use and a restricting of its performance as a photo/sonosensitizer. In fact, its tendency to aggregate in physiological environments quenches the excited states of the PS that are crucial to produce ROS, reducing the overall efficiency of Ce6 in PDT/SDT treatments.

In addition, from a translational point of view, Ce6 is characterized by a non-specific cellular uptake, a poor biodistribution, and a short circulation time in vivo, which is what determines its low tumor accumulation [8].

Several studies focused on the enhancement of the pharmacological profile of Ce6 by a bioconjugation of the molecular scaffold with targeting agents [9,10,11,12,13,14,15] or by using nano-based delivery systems [2,16] to improve its solubility and stability in physiological environments. This was in addition to investigating the pharmacokinetics and pharmacodynamics properties of the molecule.

The investigation of the interaction of Ce6 with human serum albumin (HSA) is a crucial issue that involves both the following aspects: (i) HSA is the endogenous carrier of Ce6 in the blood and the formation of this complex governs its cellular uptake and biodistribution [17,18,19,20] and (ii) HSA can be used to develop biocompatible protein-based nanoplatforms for cancer theranostics [21,22,23,24,25,26].

The clinical efficacy of a PS in PDT is determined not only by its photophysical properties, but also by its interaction with biomolecules, cells, and tissues. After administration, the bioavailability of a PS is governed by the competitive binding to serum albumin, which is the major protein in plasma. Serum albumins act as the endogenous carrier for the bulk of PS, carrying the PS into the bloodstream as a complex with the protein.

At a physiological pH, the majority of Ce6 is bound to HSA [19]. The pH affects this interaction; lowering the pH decreases the stability of the Ce6–HSA complex with a concomitant increase of Ce6 binding to LDL and membranes [19]. The formation of the Ce6@HSA complex also affects the cellular uptake of the photosensitizer [17].

Recently, albumin has received a lot of attention in order to develop innovative delivery systems as a flexible nano-carrier [21,22,23,24,25,26]. HSA can be easily obtained commercially from human serum. It is stable, biocompatible, biodegradable, non-toxic, and non-immunogenic [21,23]. HSA can target cancer cells/tissues both passively, via the enhanced permeability and retention (EPR) effect, and actively, via HSA receptors that are overexpressed by cancer cells [21,23].

All these properties make HSA an ideal candidate to develop protein-based nanoplatforms for cancer theranostics [21,22,23,24,25,26]. The versatility of HSA as a carrier for hydrophobic drugs [27,28,29] and PSs [28,30,31,32] can be exploited also for Ce6. Thus far, despite the importance of this topic, the way in which HSA binds Ce6, the atomistic details of their interactions, and the effect of the HSA on the ability to generate ROS are still not known in detail.

**Figure 1:**
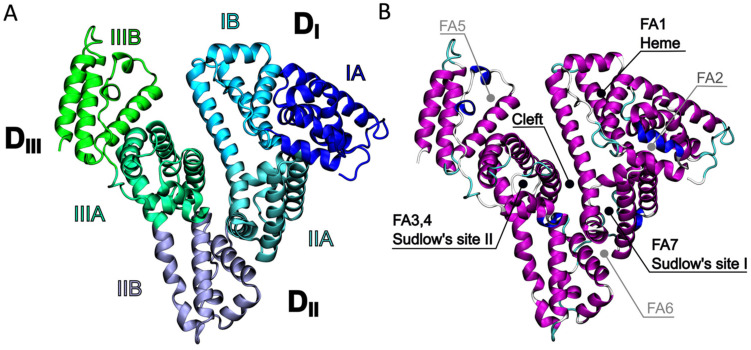
The 3D structure of HSA: (**A**) Subdivision into domains (I–III) and subdomains (A, B); (**B**) identification of the binding sites.

HSA is a heart-shaped protein with a molecular weight of about 66.5 kDa. Its structure consists of three homologous domains (DI, DII, and DIII), containing each two sub-domains (A and B), as is shown in Figure 1A. One of the main physiological roles of HSA is the transport of fatty acids (FA) in the blood and the seven distinct binding pockets for FA (FA1–7), which were identified by crystallography [33] (Figure 1B). HSA is also the carrier of many endogenous (i.e., bilirubin, thyroxine, and hemin) and exogenous (drugs) compounds [34]. Drugs are usually bound in two main binding sites [34]: Sudlow site I (FA7), located in the subdomain IIA, and Sudlow site II (FA3, FA4), located in the subdomain IIIA. Recent studies have revealed that a third important binding pocket can be identified within subdomain IB (site IB, FA1) [35], which is also the characteristic binding site of heme [36,37,38]. In addition, some PSs [31,39,40] can bind in an extra cavity in the cleft between domains DI and DIII.

Certain spectroscopic studies have clearly indicated that Ce6 binds to HSA [19,41], as well as also suggest the presence of two Ce6 binding sites [41].

Due to the structural similarity between Ce6 and heme, which is that they both possess a hydrophobic core bearing polar carboxylic chains on one side only, it was initially hypothesized that the primary binding site of heme and dicarboxylic porphyrins was the same [19], i.e., the IB site. The determination of the specific binding of Ce6 analogs, (i.e., chlorin p6 [42], purpurin 18 [42], and the iodinated chlorin p6-copper complex [43]) in the Sudlow Site I of HSA [42,43], achieved by tryptophan fluorescence quenching measurements and by a competitive binding with warfarin, suggested that Sudlow Site I is the preferred binding pocket for Ce6. Sudlow Site II was also proposed as a potential secondary site of binding for chlorins [44], even if it was characterized by a smaller binding constant than Sudlow site I.

All the studies demonstrated that the binding of Ce6 to HSA had no effect on the conformation of the protein [42,43].

## 2. Results and Discussion

### 2.1. Identification of the Ce6 Binding Pockets

**Figure 2:**
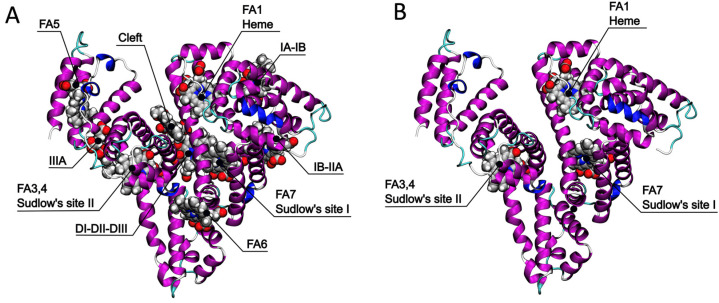
(**A**) The ten most probable Ce6 binding pockets in HSA, identified by docking; (**B**) the three most probable Ce6 binding sites in HSA identified by molecular dynamics simulations.

The possible interaction sites between Ce6 and HSA were determined by ensemble docking [45], followed by molecular dynamic (MD) simulations. By using the 129 crystallographic structures of HSA (Table S1) deposited in the protein data bank (PDB), almost two-hundred thousand poses were generated. This approach allowed a substantial sampling of the conformational space of HSA because, despite the general similarity of the HSA structures, significant domain rotations were observed upon performing the FA and ligand binding. Using a hierarchical approach, all the poses were clustered and then the ten most probable binding sites were selected using the PatchDock scoring function (Figure 2A). MD simulations of 100 ns were carried out using these ten poses as the starting geometries (Figure S1). The position of Ce6 in the various docking modes was stable (Figure S2) during MD simulations and all the interacting geometries provided favorable interactions with HSA (Figure S1). Interaction energies between Ce6 and HSA were calculated using the MM-GBSA approach. For the three most favored binding sites, the MD simulations were extended to 1 ms (Figure 2B). Very interestingly, the three putative binding sites of Ce6 proposed in the literature—i.e., the heme binding site (IB) [19], Sudlow I (SI) [42,43] and Sudlow II (SII) [44]—were identified as the most interacting ones (Figure 2B). The energetic values of the interaction suggest that the preferential site for Ce6 binding is Sudlow I (SI, ΔE_binding_ = −70.6 kcal mol^−1^), followed by the heme binding site (IB, ΔE_binding_ = −61.0 kcal mol^−1^) and Sudlow II (SII, ΔE_binding_ = −52.8 kcal mol^−1^).

This means that Sudlow I (SI) is the principal binding site for Ce6, while the heme binding site (IB) represents the secondary binding pocket. The contribution of each amino acid in the two binding pockets to the binding of Ce6 is provided by fingerprint analysis.

### 2.2. Ce6 in the Sudlow I Binding Site

**Figure 3:**
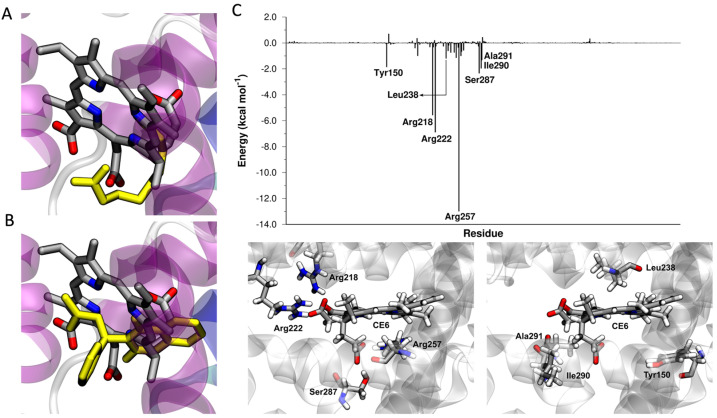
(**A**) A superposition of the crystallographic structure of myristic acid (in yellow, PDB:6YG9) and a docked structure of Ce6 in the Sudlow I site; (**B**) a superposition of the crystallographic structure of warfarin (in yellow, PDB:2BXD) and a docked structure of Ce6 in the Sudlow I site; (**C**) Ce6−HSA interactions in the Sudlow I site. ΔE_binding_ decomposed per residue. A 3D representation of the most interacting residues.

Sudlow I (SI) is a pre-formed binding pocket shared by a variety of drugs/ligands. It is located in the core of subdomain IIA and is made up of all six helices of the subdomain and a loop–helix motif (residues 148–154) from domain IB. The interior of the pocket is hydrophobic, but two clusters of positively charged residues are present on the cavity entrance. Ce6 occupies the FA7 binding pocket (Figure 3A), superimposing perfectly with the crystallographic structure of warfarin (Figure 3B), thus explaining the experimental results showing the competitive binding of chlorins with warfarin [42,43].

The ligands in Sudlow I always have a planar group/ring sandwiched between the aliphatic side chains of Leu238 or Ala291 [34]. The same is observed for the planar ring of the Ce6. Ile290, via hydrophobic interactions and Tyr150 via π-π stacking, contribute further to the binding (Figure 3C and Table S2).

Molecules bound in the Sudlow I site usually also engage interactions with the positively charged residues located at the entrance of the binding pocket [34]. Ce6 makes strong hydrogen bond/salt bridge interactions with Arg218, Arg222, Arg257, and Ser287 (Figure 3C and Table S2) by using its three carboxylic groups.

### 2.3. Ce6 in the Heme Binding Site

The heme binding site is entirely contained in the IB subdomain of HSA and is made up of four contiguous helices and a loop. The hydrophobic porphyrin ring of the heme is buried in a hydrophobic cleft created by the subdomain helices, while the propionate groups are situated near the entrance of the pocket, where they can interact with solvent molecules and a triad of positively charged residues [36,37]. The iron atom of the heme is coordinated by Tyr161 [36,37].

**Figure 4:**
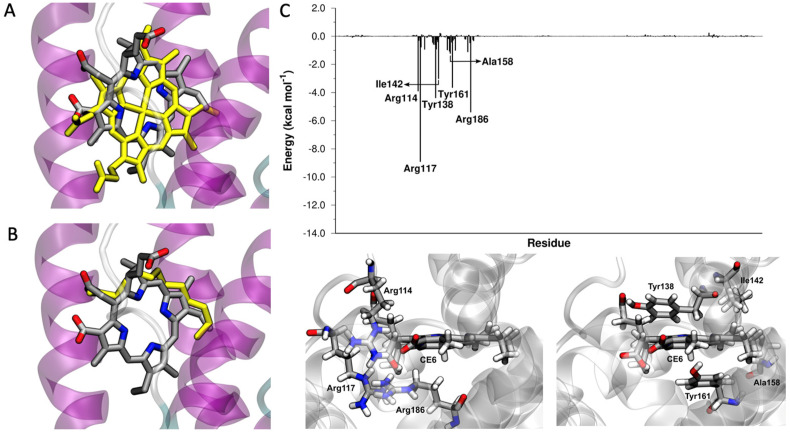
(**A**) A superposition of the crystallographic structure of heme (in yellow, PDB:1O9X) and a docked structure of Ce6 in the heme binding pocket; (**B**) a superposition of the crystallographic structure of myristic acid (in yellow, PDB:1BJ5) and a docked structure of Ce6 in the heme binding pocket; (**C**) Ce6−HSA interactions in the heme binding pocket. ΔE_binding_ decomposed per residue. A 3D representation of the most interacting residues.

Ce6 is accommodated in the same way that the heme is (Figure 4A), which is with the chlorin ring bound within the narrow hydrophobic cavity, whereby the heme ring is also bound. The rim of the chlorin ring overlaps perfectly with the curved structure adopted by the myristate (Figure 4A) when occupying this site [33,46]. The Ce6 ring is sandwiched between two tyrosine residues (Tyr161 and Tyr128) by π-π interactions. Due to the absence of the iron atom in the Ce6, Tyr161 changes its role in the interaction with Ce6 when compared to the crystallographic structure of HSA in complex with the heme. Ile142 and Ala158 also provide hydrophobic stabilization to Ce6 (Figure 4C and Table S3).

With its three carboxylic groups, Ce6 can provide even better stabilization than heme when we consider hydrogen bonds/salt bridges with the basic residues at the entrance of the pocket. In fact, three arginine residues (Arg 114, Arg 117, and Arg186) strongly interact with Ce6 (Figure 4C and Table S3). Arg117, which always interacts with the fatty acids that bind here, surprisingly does not bind the carboxylic groups of heme. However, it has a primary role in the binding with Ce6.

### 2.4. Effect of the Binding of Ce6 on the Structure of HSA

Experimentally it was found that the 3D structure of the HSA protein was not perturbed by the interaction with chlorins [42]. The analysis of the secondary structure of the Ce6@HSA complexes, during the MD simulations, showed that the 3D and the secondary structures of HSA were practically unaffected by the Ce6 binding (Table 1, Figures S3–S5).

In the crystal structure of HSA, C-terminal helices in domain III are characterized by very high temperature factors [47]. The formation of the Ce6@HSA complexes even increases the stability of these terminal helices (Table 1, Figure S3). The Rg analysis (Figure S4) and RMSD (Figure S5) of HSA and Ce6@HSA during 1-μs-long MD simulations also demonstrated that there were no changes in the tertiary structure of the protein.

### 2.5. Comparison between Experimental and Computational Results about Ce6 Binding

When comparing the binding of different porphyrins/chlorins with HSA, it was demonstrated that the interaction is stronger when these hydrophobic molecules contain a hydrophilic side [41]. The structure of the complexes between Ce6 and HSA—obtained here by docking and MD simulations—perfectly explains this behavior, showing the following: (i) the hydrophobic ring of the chlorin is accommodated in the hydrophobic pockets of Sudlow I site and of the heme binding site, thus driving the PS binding; (ii) the charged carboxylic groups, present at the rim of the Ce6 molecule, interact via the hydrogen bond/salt bridge with the positively charged residues located at the entrance of the two pockets.

In addition, if the binding constants of Ce6 and its monomethyl ester for HSA are very similar, the association constants of the dimethyl and trimethyl ester derivatives showed, respectively, a reduction of 3 and 11 times [18], thus indicating that the stability of the PS—protein complex depends on the participation of at least two negatively charged side groups. The changes in the charge of the side groups significantly affect the electrostatic interactions between Ce6 and HSA.

Interestingly, the affinity of Ce6 to HSA decreases when the pH is lowered [18,19]. Additionally, this effect can be ascribed to the three carboxylic groups of the Ce6. In particular, their protonation strongly reduces the electrostatic interactions between the PS and the protein.

The reduced stability of the Ce6@HSA complex upon a small pH decrease can also have a crucial role in the uptake of Ce6 by cancer cells. This is because it can favor the targeted release of Ce6 to cancer tissues, and is characterized by a more acidic microenvironment than what is found in normal tissues.

### 2.6. Synthesis and Characterization of Ce6@HSA

To study the effect of HSA binding on the photophysical and photosensitizing properties of Ce6, a Ce6@HSA complex—characterized by a well-defined 1:1 stoichiometry (with occupation of a single pocket, i.e., the Sudlow I and SI)—was synthesized by utilizing a PBS/DMSO mixed solvent solution.

**Figure 5:**
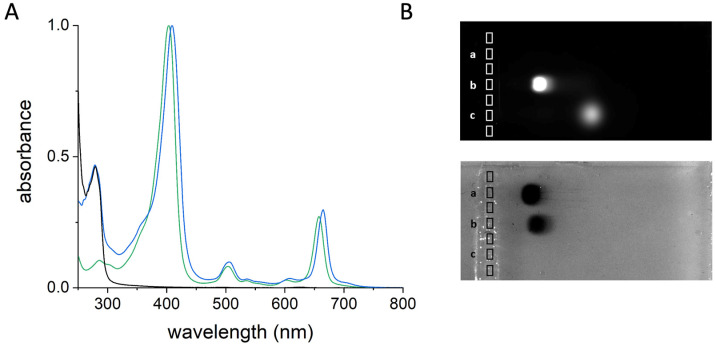
(**A**) The UV-Vis spectra of HSA (black line), Ce6 (green line), and Ce6@HSA (blue line); (**B**) the agarose gel electrophoresis of (a) HSA, (b) Ce6@HSA, and (c) Ce6. The gel images were acquired both before staining, in fluorescent mode (top), and after staining with Coomassie blue, in colorimetric mode (bottom).

The UV-Vis spectrum of Ce6@HSA (Figure 5A) suggests that Ce6 has been successfully incorporated into HSA. In fact, it showed the characteristic diagnostic bands of both the protein (at 281 nm) and the Ce6 (the Soret band at ~400 nm and the Q bands between 500 and 680 nm), which were slightly perturbed when compared to the reference of Ce6, due to the interaction with the protein.

The electrophoretic analysis of Ce6@HSA, carried out on agarose gel in native conditions, demonstrates unequivocally that Ce6 is encapsulated in the protein (line b, Figure 5B). The gel acquisition, in fluorescent mode, demonstrated that Ce6@HSA (line b, Figure 5B) does not show any signal related to the presence of free Ce6 (line c, Figure 5B). In addition, the fluorescent spot perfectly matches the spot of the protein, which is visible after the Coomassie staining, demonstrating the simultaneous presence of both HSA and Ce6. It is interesting to observe that the migration rate of the complex (line a, Figure 5B) is slightly higher than free protein (line a, Figure 5B), most likely due to the lower mass/charge ratio of the Ce6@HSA when compared to free HSA (negatively charged), which is due to the presence of the three additional negative charges of the Ce6 in the complex.

### 2.7. Photophysical Properties of Ce6@HSA

The Ce6@HSA adduct presents noticeable changes in the photophysical properties of the Ce6 (Table 2).

**Figure 6:**
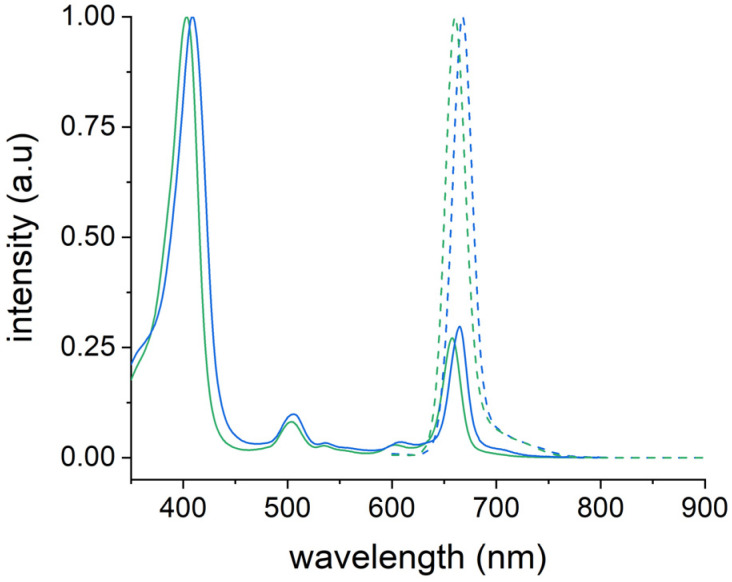
Normalized absorption (full line) and fluorescence (l_ex_ = 500 nm, dashed line) spectra of Ce6 (green lines) and Ce6@HSA (blue lines) in PBS at room temperature.

In particular, in PBS, a 6 nm red-shift can be observed in both the absorption and emission spectra of Ce6@HSA (Figure 6) when compared to the spectra of Ce6. Additionally, the strongest of the Q bands, which is the most important from a translational point of view since it is located in the transparency window, features an even larger red-shift of 8 nm. Moreover, the ratio between the Soret band and the Q band at 667 was lower in Ce6@HSA than in Ce6.

Ce6@HSA maintains the fluorescence quantum yield of the free Ce6, i.e., 0.17, but shows an increase of the excited state lifetime from 4.5 to 5.2 ns. The red-shifts observed in both the absorption and emission spectra, as well as in the extension of the lifetime, supported the encapsulation of Ce6 inside HSA.

### 2.8. Generation of ROS by Ce6@HSA

When the light is adsorbed by a PS, ROS can be produced following two different mechanisms. In the type I mechanism, the excited PS is involved in a hydrogen or electron transfer process to form a radical, which reacts with water or molecular oxygen, producing different ROS, such as superoxide anions, hydroxyl radicals, and hydrogen peroxide. In the type II mechanism, the excited PS directly transfers its energy to ground state oxygen ^3^O_2_ to generate a singlet oxygen excited state (^1^O_2_). The amount of peroxides and ^1^O_2_ generated during visible light irradiation via type I and type II mechanisms were estimated by using the Amplex Red [48,49,50] and the ABMDMA [48,50] assays, respectively.

**Figure 7:**
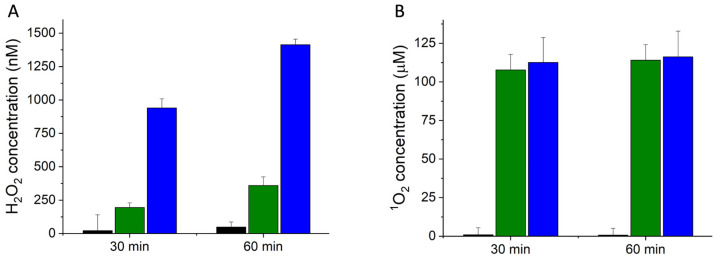
Quantification of ROS generated through type I (**A**) and type II (**B**) mechanisms at different irradiation times (30 min and 60 min), white light irradiation by Ce6 (green bars), Ce6@HSA (blue bars), and PBS (control, black bars). (**A**) Amplex Red assay for peroxides detection; (**B**) ABMDMA assay for ^1^O_2_ detection.

The results (Figure 7) showed that the encapsulation of Ce6 in HSA does not affect significatively the type II mechanism. On the opposite end, the type I mechanism is strongly improved (~400%). The type I mechanism is often activated by sacrificial electron donors, in fact, electron-rich environments boost photoactivation and favor the type I over type II mechanism [51].

As the protein residues in HSA may take part in the electron transfer activities directly, Ce6@HSA does not require any external electron donors. This indicates that the presence of the protein itself induces a self-activation of the type I mechanism [22,50,52,53,54,55,56,57] in the Ce6@HSA adduct.

This aspect is extremely interesting from an applicative point of view because, due to their reduced reliance on oxygen content, PSs producing ROS via the type I mechanism are increasingly being preferred in anticancer PDT [58,59,60] because they can overcome the hypoxic milieu found in tumor tissue.

## 3. Materials and Methods

### 3.1. Computational Analysis of the Ce6@HSA Complex

#### 3.1.1. Human Serum Albumin Structural Database

Human serum albumin (HSA) crystal structures were downloaded from the Protein Data Bank (PDB) [61]. A total of 129 PDB files were obtained and processed by removing water molecules, ions, and co-crystallized ligands. This dataset was used for ensemble docking calculations using the Chlorin e6 (Ce6) structure as a ligand.

#### 3.1.2. Docking

Ensemble docking calculations were carried out using the Ce6 ligand and for every HSA PDB structure. The docking poses were obtained using the PatchDock algorithm [62], following a global search. PatchDock carries out a rigid docking, maximizing the surface shape complementarity between the protein receptor and the ligand. The algorithm implicitly addresses surface flexibility by using soft potentials that allow for small atomic compenetrations.

The scoring function then assigns a geometric shape complementarity score that evaluates the interface area and the desolvation energy associated with the protein-ligand interactions. These terms describe accurately the binding of proteins with rigid and hydrophobic moieties, such as chlorin, which are already demonstrated for fullerenes [63,64,65,66,67,68,69], carbon nanotubes [70,71,72,73], and carboranes [74].

All the docking poses were then clustered, and the ten most probable binding sites were selected using the PatchDock scoring functions [62].

#### 3.1.3. Minimization and MD Simulations

The Amber ff14SB force field [75] was used to model the HSA. Ce6 atoms were modeled using the GAFF force field. The atomic charges of the Ce6 atoms were determined using the Merz-Singh-Kollman scheme. The corresponding parameters for Ce6 were generated by the standard procedure that is reported for an antechamber, as implemented in Amber 16 [76]. All simulations and minimization were performed using the TIP3P water model, and sodium counterions were added to maintain the electric neutrality of the system. Periodic boundary conditions (PBC) and the particle mesh Ewald summation were used throughout (with a cut-off radius of 10.0 Å). H-atoms were considered using the SHAKE algorithm and a time step of 2 fs was set during all the MD runs. A total of 5000 steps of a steepest descent minimization, followed by an additional 5000 steps of conjugate gradient minimization, were performed with PMEMD [76]. The minimized structures were subjected to an equilibration process (individual equilibration steps included (i) 500 ps of heating from 0 to 298 K within an NVT ensemble and (ii) 4500 ps of equilibration MD at 298 K to switch from NVT to NPT and to adjust the simulation box; isotropic position scaling was used at the default conditions; and the Langevin temperature equilibration scheme (ntt = 3) was used). Then, 100 ns/1 μs of MD simulations were carried out. Snapshot structures were saved into individual trajectory files every 1000-time steps, i.e., every 2 ps of the MD simulation.

The secondary structure analyses of HSA and Ce6@HSA were carried out by using a VMD timeline. The radius of gyration (R_g_) and the root-mean-square deviation of the atomic positions during MD simulations were calculated by CPPTRAJ [77].

#### 3.1.4. Molecular Mechanics/Generalized Born Surface Area (MM/GBSA) Analysis

One frame every 0.1 ns was extracted from the MD trajectory by means of CPPTRAJ [77], and used as input for the MM-GBSA analysis to calculate the binding affinity between Ce6 and HSA. An infinite cut-off was used for all the interactions. The electrostatic contribution to the solvation free energy was calculated using the generalized Born (GB) model (igb = 5), as implemented in MMPBSA.py [78]. The non-polar contribution to the solvation free energy was determined using solvent-accessible, surface-area-dependent terms. The per-residue decomposition of ΔE_binding_ (fingerprint analysis) was obtained by MMPBSA.py [78].

### 3.2. Synthesis and Characterization of the Ce6@HSA Complex

#### 3.2.1. Materials

Human serum albumin fatty acid free (HSA) (Cat. No. A3782); dimethyl sulfoxide (DMSO) (Cat. No. 472301); deuterium oxide (Cat. No. 151882-100G); 9,10-anthracenediylbis(methylene)dimalonic acid (ABMDMA) (Cat. No. 75068); 10-Acetyl-3,7-dihydroxyphenoxazine (Amplex Red) (Cat. No. 90101); Type VI-A Peroxidase from horseradish lyophilized powder (HRP) (Cat. No. P6782); Hydrogen Peroxide Solution 30% (*w*/*w*) (Cat. No. 31642-M); Amicon Ultra centrifugal filters (MWCO 30 kDa, Millipore UFC503024, Cat. No. Z677892-24EA); sodium chloride (Cat. No. S9888-M); potassium phosphate monobasic (Cat. No. P0662-M); sodium phosphate dibasic (Cat. No. S0876); potassium chloride (Cat. No. P3911M); and MWCO 14 kDa dialysis tubing cellulose membrane (Cat. No. D9652) were all purchased from Sigma Aldrich (Merck, Darmstadt, Germany). Chlorin e6 (Item No. 21684) was purchased from Cayman Chemical (Ann Arbor, MI, USA). All the reagents were used without further purifications. Milli-Q water was used for the preparation of all the aqueous solutions.

#### 3.2.2. Synthesis and Purification of the Ce6@HSA Complex

The complex Ce6@HSA was synthesized by using a procedure that was recently developed to encapsulate hydrophobic PS inside HSA [31]. Ce6 and HSA were used in 1:1 stoichiometry. Briefly, 500 µL of a solution of Ce6 in PBS/DMSO (5/3 *v*/*v*) was prepared at a concentration of 200 µM. It was then added to 500 µL of an equimolar solution of HSA, which was previously dissolved in the same mixture of PBS/DMSO (5/3 *v*/*v*).

The mixture containing 100 µM of both the components was then incubated overnight at 25 °C under continuous shaking at 700 rpm (ThermoMixer HC, S8012-0000; STARLAB, Hamburg, Germany). The mixture was than dialyzed in PBS, using a MWCO 14 kDa dialysis tubing cellulose membrane, in order to remove free Ce6 and DMSO. The purified solution was then analyzed by UV-Vis spectroscopy, showing a final stoichiometry of 0.5:1 of Ce6/HSA.

#### 3.2.3. Characterization of the Ce6@HSA Complex

*UV-Vis Spectroscopy.* Ce6, HSA, and Ce6@HSA were characterized through UV-Vis spectroscopy. The spectroscopic data were collected using a Cary60 UV-Vis spectrophotometer (Agilent Technologies, Stockport, UK).

*Fluorescence Spectroscopy.* The emission spectra and the excited state lifetime of Ce6 and Ce6@HSA were acquired with an Edinburgh FLS920, which was equipped with a photomultiplier Hamamatsu R928P. The fluorescence quantum yields were measured by taking the tetraphenylporphyrin (TPP) in toluene as standard [79].

*Agarose gel electrophoresis.* Ce6, HSA, and Ce6@HSA were characterized by agarose gel electrophoresis, in native conditions (Owl Easycast B-Series Horizontal Gel Systems Model B2). Further, 1% *w/v* concentration of gel was prepared, dissolving the agarose powder in a tris-glycine buffer at pH 7.4. Moreover, 20% *v*/*v* of glycerol was added to each sample, and 12 µL of the mixture was loaded into each well. Then, 15 µg of protein was loaded into the wells, and a solution of Ce6 was loaded separately as a reference. The run was performed by applying a voltage of 100 V for 30 min and tris-glycine pH 7.4 was used as the running buffer.

The gel was acquired using a ChemiDoc MP Imager, both in colorimetric and fluorometric modes (Ex. Alexa 647).

#### 3.2.4. Detection of Reactive Oxygen Species

*ABMDMA assay.* This colorimetric assay selectively detects and estimates the amount of singlet oxygen (^1^O_2_) in solution. The assay is based on the decrease of the UV absorption band of ABMDMA when it reacts with singlet oxygen [31,48,50].

The samples (Ce6 and Ce6@HSA) were used in deuterated PBS. Then, 3 µL of an ABMDMA 5 mM stock solution in DMSO was added to 97 µL of each sample that was loaded into the wells of a 96 multi-well plate. The plate was exposed to a light source (Valex 30 W, 6500 K, cold white LED), positioned at 30 cm distance from the cell plate surface (irradiance = 1.5 mW cm^−2^, energy fluence = 2.7 and 5.4 J cm^−2^, for 30 and 60 min of irradiation, respectively). The irradiance was measured with the photo-radiometer Delta Ohm LP 471 RAD.

*Amplex Red assay.* The Amplex Red assay allowed the quantification of peroxides in a solution. It was based on the enzymatic reaction and catalyzed by a horseradish peroxidase (HRP) that occurs between the colorless Amplex Red with peroxides, producing a pink-colored resorufin [48,49,50].

A working solution (WS), containing Amplex Red and HRP dissolved in PB, was freshly prepared. Further, 90 µL of each sample were loaded into the wells of a 96-multiwell plate. One plate was irradiated, following the same conditions of the ABMDMA assay, while an identical plate was kept in the dark. Then, 10 µL of the WS were then added to each sample and both the plates were kept in incubation for 30 min in dark conditions at room temperature.

The absorbance of the resorufin produced was recorded at 560 nm. To convert the absorbance values to the equivalent H_2_O_2_ concentration, a calibration curve was created using standard solutions of H_2_O_2_. The contribution of the H_2_O_2_ produced by the samples kept in the dark was subtracted from the H_2_O_2_ concentration that was estimated for the corresponding irradiated samples. All the measurements were performed using an EnSpire^®^ Multimode Plate Reader (PerkinElmer).

## Figures and Tables

**Table 1:** Percentage of the secondary structure in HSA during 1 μs MD simulations for HSA, Ce6@HSA (SI site), and Ce6@HSA (Heme site).

Secondary Structures	HSA	Ce6@HSA (SI Site)	Ce6@HSA (Heme Site)
Coil	13.1	13.0	12.9
π Helix	0.0	0.1	0.1
3–10 Helix	2.9	3.6	3.3
α Helix	65.6	68.8	68.4
Isolated β Bridge	0.2	0.0	0.0
β Sheet	0.0	0.0	0.0
Turn	18.3	14.6	15.3

**Table 2:** Main photophysical parameters of Ce6 and Ce6@HSA in PBS at room temperature.

	Absorption		Emission		
	λ_max,Soret_ (nm)	λ_max,Q-band_ (nm)	λ_max_ (nm)	Φ	t (ns)
Ce6	403	657	661	0.17	4.5
Ce6@HSA	409	665	667	0.17	5.2

## Data Availability

All data in this study can be requested from the corresponding authors (matteo.digiosia2@unibo.it, M.D. and matteo.calvaresi3@unibo.it, M.C.).

## Supplementary Materials

The following supporting information can be downloaded at https://www.mdpi.com/article/10.3390/molecules28052348/s1, Table S1: Human serum albumin structural database; Figure S1: ΔE_binding_ of Ce6 and HSA in the ten most probable binding pockets of HSA; Figure S2: Ce6 RMSD vs. time analysis in the docked poses; Table S2: Ce6–HSA interactions in the Sudlow I site; Table S3: Ce6–HSA interactions in the heme binding pocket; Figure S3: Secondary structure analysis of HSA and Ce6@HSA during MD simulations; Figure S4: Radius of gyration of HSA and Ce6@HSA during MD simulations; and Figure S5: Protein RMSD, domain I, II, and III RMSD, Ce6 RMSD in HSA and Ce6@HSA during MD simulations.
