# Supplementary material for: Dissecting the Interactions between Chlorin e6 and Human Serum Albumin
Source: Molecules. 2023 Mar 3;28(5):2348. doi: 10.3390/molecules28052348 (PMC10005744; doi:10.3390/molecules28052348)
Supplement: Supplementary file 1 [file molecules-28-02348-s001.zip › molecules-2222402-supplementary.pdf]

Supplementary Materials

Dissecting the Interactions between Chlorin e6 and Human Serum Albumin

Alessia Marconi †, Edoardo Jun Mattioli †, Filippo Ingargiola, Giulia Giugliano, Tainah Dorina Marforio, Luca Prodi, Matteo Di Giosia * and Matteo Calvaresi *

Dipartimento di Chimica "Giacomo Ciamician", Alma Mater Studiorum—Università di Bologna, Via Francesco Selmi 2, 40126 Bologna, Italy

* Correspondence: matteo.digiosia2@unibo.it (M.D.G.); matteo.calvaresi3@unibo.it (M.C.)

† These authors contributed equally to this work.

Table S1. Human Serum Albumin Structural Database

PDB ID	Mutations	Method	Resolution (Å)	RMSD[1]
1ao6	No	X-ray	2.50	2.02
1bj5	No	X-ray	2.50	0.81
1bke	No	X-ray	3.15	3.12
1bm0	No	X-ray	2.50	2.69
1e78	No	X-ray	2.60	2.68
1e7a	No	X-ray	2.20	2.04
1e7b	No	X-ray	2.38	2.02
1e7c	No	X-ray	2.40	0.79
1e7e	No	X-ray	2.50	0.77
1e7f	No	X-ray	2.43	0.73
1e7g	No	X-ray	2.50	0.90
1e7h	No	X-ray	2.43	0.87
1e7i	No	X-ray	2.70	0.80
1gni	No	X-ray	2.40	0.75
1gnj	No	X-ray	2.60	0.85
1h9z	No	X-ray	2.50	0.71
1ha2	No	X-ray	2.50	0.71
1hk1	No	X-ray	2.65	1.92
1hk2	Yes	X-ray	2.80	1.99
1hk3	Yes	X-ray	2.80	1.90
1hk4	No	X-ray	2.40	0.73
1hk5	Yes	X-ray	2.70	0.81
1n5u	No	X-ray	1.90	0
1o9x	No	X-ray	3.20	1.66
1tf0	No	X-ray	2.70	1.96
1uor	No	X-ray	2.80	2.11
2bx8	No	X-ray	2.70	2.07
2bxa	No	X-ray	2.35	2.08
2bxb	No	X-ray	3.20	2.06
2bxc	No	X-ray	3.10	2.07
2bxd	No	X-ray	3.05	2.05
2bxe	No	X-ray	2.95	2.13
2bxf	No	X-ray	2.95	2.06
2bxg	No	X-ray	2.70	2.07
2bxh	No	X-ray	2.25	1.98
2bxi	Yes	X-ray	2.50	0.80
2bxk	Yes	X-ray	2.40	0.68
2bxl	No	X-ray	2.60	0.92
2bxm	No	X-ray	2.50	0.68
2bxn	No	X-ray	2.65	0.68
2bxo	No	X-ray	2.60	0.71
2bxp	No	X-ray	2.30	0.58
2bxq	No	X-ray	2.60	0.69
2i2z	No	X-ray	2.70	0.76
2i30	No	X-ray	2.90	0.74
2vdb	No	X-ray	2.52	1.42
2vue	No	X-ray	2.42	2.06
2vuf	No	X-ray	3.05	1.96

2xsi	Yes	X-ray	2.70	0.74
2xvq	No	X-ray	2.90	2.08
2xvu	No	X-ray	2.60	2.01
2xvv	Yes	X-ray	2.40	0.79
2xvw	Yes	X-ray	2.65	0.67
2xw0	No	X-ray	2.40	2.07
2xw1	No	X-ray	2.50	2.07
2ydf	No	X-ray	2.75	2.09
3a73	No	X-ray	2.19	0.97
3b9l	No	X-ray	2.60	0.78
3b9m	No	X-ray	2.70	0.73
3cx9	No	X-ray	2.80	0.60
3jry	No	X-ray	2.30	1.91
3lu6	No	X-ray	2.70	2.09
3lu7	No	X-ray	2.80	2.16
3lu8	No	X-ray	2.60	1.96
3sqj	No	X-ray	2.05	0.56
3tdl	No	X-ray	2.60	1.27
3uiv	No	X-ray	2.20	0.60
3uiv_2	No	X-ray	2.20	0.67
4bke	No	X-ray	2.35	0.82
4e99	No	X-ray	2.30	2.20
4emx	No	X-ray	2.30	2.02
4g03	No	X-ray	2.22	2.02
4g04	No	X-ray	2.30	2.03
4hgk	No	X-ray	3.04	1.69
4hgm	No	X-ray	2.34	1.69
4iw1	No	X-ray	2.56	1.99
4iw2	No	X-ray	2.41	2.01
4k2c	No	X-ray	3.23	2.04
4k71	Yes	X-ray	2.40	2.30
4l8u	No	X-ray	2.01	0.29
4l9k	No	X-ray	2.40	2.03
4l9q	No	X-ray	2.70	2.01
4la0	No	X-ray	2.40	2.02
4lb2	No	X-ray	2.80	1.99
4lb9	No	X-ray	2.70	1.22
4n0f	No	X-ray	3.02	2.18
4n0u	No	X-ray	3.80	1.91
4n0u_2	No	X-ray	3.80	1.91
4s1y	No	X-ray	3.16	1.90
4z69	No	X-ray	2.19	0.60
5fuo	No	X-ray	3.60	1.85
5gix	No	X-ray	2.80	0.65
5giy	No	X-ray	2.54	0.62
5id7	No	X-ray	2.26	1.45
5ifo	No	X-ray	3.20	0.78
5ijf	No	X-ray	2.65	2.01
5ujb	No	X-ray	2.70	1.97
5vnw	No	X-ray	2.60	1.48
5x52	No	X-ray	3.00	2.03

5yb1	No	X-ray	2.62	0.62
5yoq	No	X-ray	2.65	2.03
6a7p	No	X-ray	2.28	2.12
6ezq	No	X-ray	2.39	1.98
6hsc	No	X-ray	1.90	0.63
6je7	No	X-ray	3.90	1.59
6l4k	No	X-ray	2.09	0.59
6m58	No	X-ray	2.95	2.10
6m5d	Yes	X-ray	2.60	2.06
6m5e	No	X-ray	2.80	1.99
6m5e_2	No	X-ray	2.80	1.98
6m5e_3	No	X-ray	2.80	1.97
6qio	Yes	X-ray	1.95	2.12
6qip	Yes	X-ray	2.45	2.10
6r7s	No	X-ray	2.21	2.10
6wuw	No	X-ray	2.20	0.70
6xv0	No	X-ray	3.00	0.75
6yg9	No	X-ray	1.89	0.63
7d6j	No	X-ray	3.29	2.07
7jwn	No	X-ray	2.60	1.29
6m4r	No	X-ray	2.49	1.98
6zl1	Yes	X-ray	3.27	1.50
7a9c	Yes	X-ray	2.75	1.13
7aae	No	X-ray	2.27	0.61
7aai	No	X-ray	2.10	0.78
7djn	No	X-ray	2.04	2.16
7dl4	No	X-ray	2.40	0.48
7ffr	No	X-ray	2.31	1.94
7ov1	No	X-ray	1.90	0.65
7ov5	No	X-ray	1.90	0.80

[1] The RMSD value is calculated towards the pdb structure characterized by the best resolution, i.e. 1n5u. The RMSD value is calculated taking into account only the Cα of the proteins.

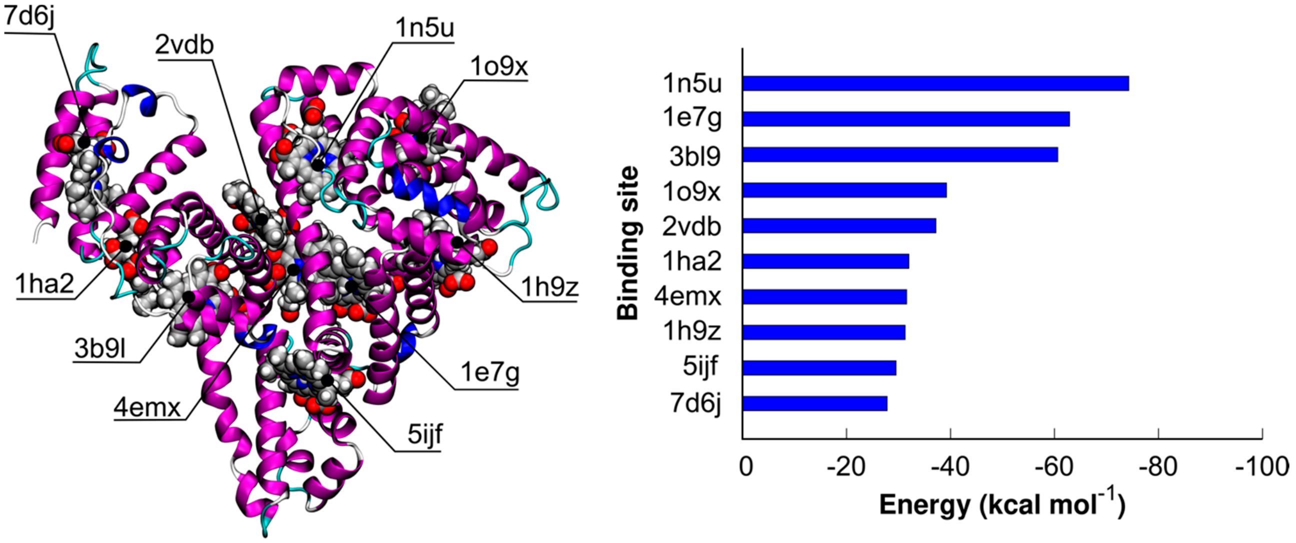

Figure S1. $\Delta E_{binding}$ of Ce6 and HSA in the ten most probable binding pockets of HSA (the PDB code of the crystallographic structure of HSA used as receptor is indicated in the figure). The $\Delta E_{binding}$ values were obtained by MM-GBSA calculations on 100 ns-long MD trajectories (one for complex).

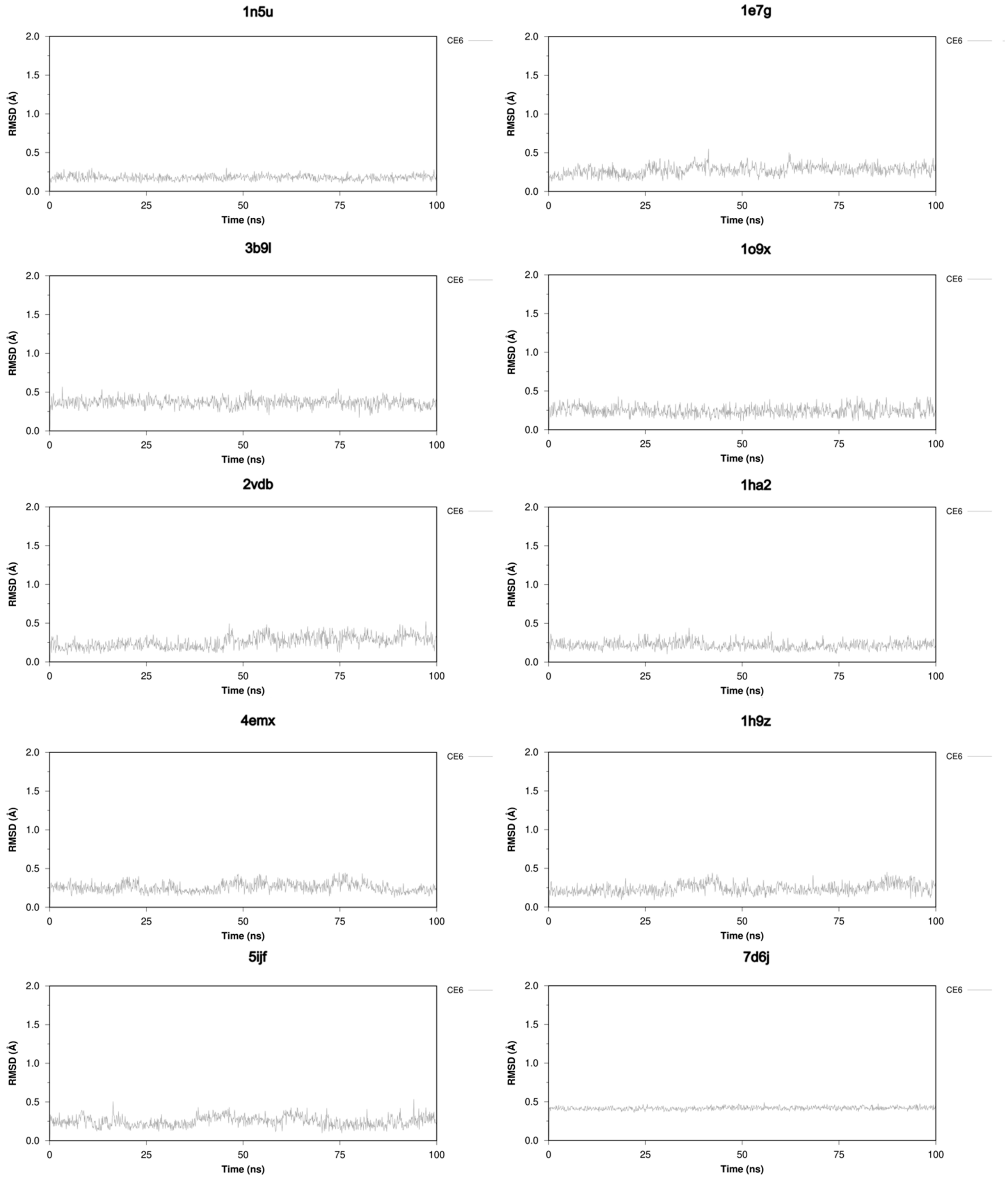

PDB	1n5u	1e7g	3b9l	1o9x	2vdb	1ha2	4emx	1h9z	5ijf	7d6j
**RMSD**	0.2	0.3	0.4	0.2	0.3	0.2	0.3	0.2	0.3	0.4

Figure S2. RMSD of Ce6 in the ten most probable binding pockets of HSA during the 100 ns MD simulations.

Table S2. Ce6-HSA interactions in the Sudlow I site. $\Delta E_{binding}$ decomposed per residue. Energetic value of the interactions and Std Dev.

ASP	1	0.007	+/-	0.020
ALA	2	0.000	+/-	0.002
HIE	3	0.002	+/-	0.003
LYS	4	-0.023	+/-	0.008
SER	5	0.012	+/-	0.006
GLU	6	0.087	+/-	0.016
VAL	7	0.024	+/-	0.009
ALA	8	0.007	+/-	0.005
HIE	9	0.034	+/-	0.012
ARG	10	0.079	+/-	0.062
PHE	11	0.000	+/-	0.007
LYS	12	-0.026	+/-	0.007
ASP	13	0.138	+/-	0.029
LEU	14	0.019	+/-	0.009
GLY	15	-0.008	+/-	0.005
GLU	16	0.033	+/-	0.004
GLU	17	0.039	+/-	0.007
ASN	18	0.049	+/-	0.012
PHE	19	-0.009	+/-	0.007
LYS	20	-0.026	+/-	0.005
ALA	21	0.018	+/-	0.007
LEU	22	0.016	+/-	0.014
VAL	23	0.014	+/-	0.006
LEU	24	0.012	+/-	0.005
ILE	25	0.009	+/-	0.009
ALA	26	0.008	+/-	0.004
PHE	27	-0.006	+/-	0.003
ALA	28	0.010	+/-	0.003
GLN	29	0.023	+/-	0.011
TYR	30	-0.018	+/-	0.006
LEU	31	0.004	+/-	0.002
GLN	32	0.006	+/-	0.003
GLN	33	-0.001	+/-	0.001
CYS	34	0.002	+/-	0.001
PRO	35	0.000	+/-	0.001
PHE	36	-0.002	+/-	0.001
GLU	37	0.012	+/-	0.001
ASP	38	0.013	+/-	0.001
HIE	39	0.001	+/-	0.002
VAL	40	0.001	+/-	0.001
LYS	41	-0.010	+/-	0.001
LEU	42	0.006	+/-	0.002
VAL	43	0.007	+/-	0.003
ASN	44	0.001	+/-	0.001
GLU	45	0.017	+/-	0.002
VAL	46	0.014	+/-	0.004
THR	47	0.000	+/-	0.002
GLU	48	0.015	+/-	0.002
PHE	49	0.003	+/-	0.002
ALA	50	0.002	+/-	0.002
LYS	51	-0.024	+/-	0.003
THR	52	0.000	+/-	0.001
CYX	53	0.001	+/-	0.002
VAL	54	-0.001	+/-	0.001
ALA	55	-0.001	+/-	0.001
ASP	56	0.013	+/-	0.002

GLU	57	0.019	+/-	0.008
SER	58	-0.000	+/-	0.001
ALA	59	0.001	+/-	0.001
GLU	60	0.015	+/-	0.002
ASN	61	0.005	+/-	0.003
CYX	62	0.004	+/-	0.002
ASP	63	0.038	+/-	0.010
LYS	64	-0.001	+/-	0.009
SER	65	-0.002	+/-	0.012
LEU	66	0.021	+/-	0.011
HIE	67	0.003	+/-	0.009
THR	68	0.022	+/-	0.010
LEU	69	0.015	+/-	0.006
PHE	70	-0.008	+/-	0.004
GLY	71	0.002	+/-	0.003
ASP	72	0.019	+/-	0.003
LYS	73	-0.015	+/-	0.003
LEU	74	0.007	+/-	0.003
CYX	75	-0.004	+/-	0.001
THR	76	-0.001	+/-	0.001
VAL	77	0.001	+/-	0.001
ALA	78	-0.000	+/-	0.001
THR	79	0.001	+/-	0.001
LEU	80	0.002	+/-	0.001
ARG	81	-0.010	+/-	0.001
GLU	82	0.007	+/-	0.001
THR	83	0.001	+/-	0.001
TYR	84	-0.002	+/-	0.002
GLY	85	0.000	+/-	0.001
GLU	86	0.014	+/-	0.003
MET	87	0.003	+/-	0.002
ALA	88	-0.001	+/-	0.001
ASP	89	0.011	+/-	0.001
CYX	90	0.005	+/-	0.002
CYX	91	-0.001	+/-	0.001
ALA	92	-0.000	+/-	0.001
LYS	93	-0.013	+/-	0.001
GLN	94	0.003	+/-	0.002
GLU	95	0.047	+/-	0.014
PRO	96	-0.003	+/-	0.004
GLU	97	0.033	+/-	0.008
ARG	98	-0.004	+/-	0.006
ASN	99	0.014	+/-	0.013
GLU	100	0.049	+/-	0.011
CYX	101	0.013	+/-	0.005
PHE	102	0.001	+/-	0.003
LEU	103	0.008	+/-	0.006
GLN	104	0.003	+/-	0.004
HIE	105	0.001	+/-	0.003
LYS	106	0.007	+/-	0.017
ASP	107	0.031	+/-	0.008
ASP	108	0.053	+/-	0.017
ASN	109	0.013	+/-	0.007
PRO	110	-0.002	+/-	0.004
ASN	111	0.005	+/-	0.006
LEU	112	0.002	+/-	0.002
PRO	113	-0.003	+/-	0.002
ARG	114	-0.012	+/-	0.004
LEU	115	0.000	+/-	0.001
VAL	116	0.001	+/-	0.001

ARG	117	-0.017	+/-	0.003
PRO	118	-0.001	+/-	0.001
GLU	119	0.009	+/-	0.001
VAL	120	0.001	+/-	0.001
ASP	121	0.008	+/-	0.001
VAL	122	0.000	+/-	0.001
MET	123	0.001	+/-	0.001
CYX	124	0.000	+/-	0.001
THR	125	-0.000	+/-	0.001
ALA	126	0.001	+/-	0.001
PHE	127	-0.001	+/-	0.001
HIE	128	-0.001	+/-	0.001
ASP	129	0.010	+/-	0.001
ASN	130	0.002	+/-	0.001
GLU	131	0.025	+/-	0.005
GLU	132	0.040	+/-	0.009
THR	133	0.002	+/-	0.001
PHE	134	0.003	+/-	0.001
LEU	135	0.009	+/-	0.004
LYS	136	-0.021	+/-	0.005
LYS	137	-0.017	+/-	0.002
TYR	138	0.007	+/-	0.003
LEU	139	0.013	+/-	0.005
TYR	140	-0.001	+/-	0.002
GLU	141	0.020	+/-	0.004
ILE	142	0.013	+/-	0.005
ALA	143	0.013	+/-	0.004
ARG	144	-0.015	+/-	0.003
ARG	145	-0.006	+/-	0.005
HIE	146	0.017	+/-	0.012
PRO	147	0.019	+/-	0.007
TYR	148	0.004	+/-	0.014
PHE	149	-0.073	+/-	0.025
TYR	150	-1.853	+/-	0.525
ALA	151	-0.006	+/-	0.030
PRO	152	-0.049	+/-	0.061
GLU	153	0.701	+/-	0.503
LEU	154	0.029	+/-	0.018
LEU	155	0.058	+/-	0.024
PHE	156	-0.116	+/-	0.088
PHE	157	-0.030	+/-	0.016
ALA	158	0.007	+/-	0.006
LYS	159	-0.033	+/-	0.029
ARG	160	-0.065	+/-	0.027
TYR	161	-0.005	+/-	0.004
LYS	162	-0.037	+/-	0.004
ALA	163	-0.003	+/-	0.001
ALA	164	0.001	+/-	0.001
PHE	165	-0.005	+/-	0.001
THR	166	-0.002	+/-	0.001
GLU	167	0.024	+/-	0.005
CYX	168	-0.001	+/-	0.001
CYX	169	-0.001	+/-	0.001
GLN	170	-0.001	+/-	0.001
ALA	171	-0.000	+/-	0.001
ALA	172	-0.000	+/-	0.001
ASP	173	0.009	+/-	0.002
LYS	174	-0.008	+/-	0.001
ALA	175	0.001	+/-	0.001
ALA	176	0.001	+/-	0.001

CYX	177	0.003	+/-	0.001
LEU	178	0.004	+/-	0.001
LEU	179	0.003	+/-	0.001
PRO	180	0.001	+/-	0.001
LYS	181	-0.022	+/-	0.004
LEU	182	0.007	+/-	0.002
ASP	183	0.026	+/-	0.003
GLU	184	0.064	+/-	0.011
LEU	185	0.019	+/-	0.005
ARG	186	-0.016	+/-	0.008
ASP	187	0.037	+/-	0.010
GLU	188	0.157	+/-	0.044
GLY	189	-0.019	+/-	0.013
LYS	190	0.007	+/-	0.018
ALA	191	-0.014	+/-	0.033
SER	192	-0.393	+/-	0.182
SER	193	-0.091	+/-	0.040
ALA	194	-0.006	+/-	0.034
LYS	195	0.359	+/-	0.605
GLN	196	-0.988	+/-	0.289
ARG	197	0.065	+/-	0.050
LEU	198	0.057	+/-	0.053
LYS	199	-0.117	+/-	2.025
CYX	200	-0.028	+/-	0.067
ALA	201	0.032	+/-	0.014
SER	202	0.015	+/-	0.030
LEU	203	-0.031	+/-	0.024
GLN	204	0.037	+/-	0.014
LYS	205	-0.015	+/-	0.011
PHE	206	-0.019	+/-	0.014
GLY	207	0.013	+/-	0.007
GLU	208	0.077	+/-	0.010
ARG	209	-0.024	+/-	0.007
ALA	210	0.027	+/-	0.012
PHE	211	-0.074	+/-	0.044
LYS	212	-0.001	+/-	0.026
ALA	213	0.034	+/-	0.019
TRP	214	-0.314	+/-	0.311
ALA	215	-0.078	+/-	0.054
VAL	216	0.050	+/-	0.034
ALA	217	0.026	+/-	0.055
ARG	218	-5.566	+/-	2.791
LEU	219	-0.326	+/-	0.183
SER	220	0.075	+/-	0.030
GLN	221	0.200	+/-	0.059
ARG	222	-6.885	+/-	2.131
PHE	223	-0.251	+/-	0.061
PRO	224	-0.045	+/-	0.016
LYS	225	-0.057	+/-	0.009
ALA	226	0.020	+/-	0.009
GLU	227	0.052	+/-	0.010
PHE	228	-0.011	+/-	0.008
ALA	229	0.013	+/-	0.007
GLU	230	0.105	+/-	0.024
VAL	231	0.023	+/-	0.025
SER	232	-0.012	+/-	0.014
LYS	233	0.029	+/-	0.033
LEU	234	-0.253	+/-	0.163
VAL	235	-0.024	+/-	0.029
THR	236	0.021	+/-	0.022

ASP	237	0.062	+/-	0.044
LEU	238	-1.187	+/-	0.345
THR	239	-0.027	+/-	0.026
LYS	240	0.024	+/-	0.039
VAL	241	-0.608	+/-	0.201
HIE	242	-0.185	+/-	0.544
THR	243	0.062	+/-	0.029
GLU	244	0.040	+/-	0.039
CYX	245	-0.737	+/-	0.218
CYX	246	0.052	+/-	0.058
HIE	247	0.030	+/-	0.017
GLY	248	0.016	+/-	0.014
ASP	249	-0.022	+/-	0.063
LEU	250	-0.734	+/-	0.340
LEU	251	-0.092	+/-	0.073
GLU	252	-0.019	+/-	0.093
CYX	253	-1.137	+/-	0.383
ALA	254	-0.055	+/-	0.078
ASP	255	0.007	+/-	0.074
ASP	256	-0.343	+/-	0.219
ARG	257	-12.985	+/-	2.321
ALA	258	-0.031	+/-	0.095
ASP	259	0.058	+/-	0.069
LEU	260	-0.993	+/-	0.300
ALA	261	-0.656	+/-	0.287
LYS	262	0.056	+/-	0.063
TYR	263	0.089	+/-	0.049
ILE	264	-0.568	+/-	0.311
CYX	265	0.001	+/-	0.032
GLU	266	0.091	+/-	0.021
ASN	267	0.100	+/-	0.038
GLN	268	0.063	+/-	0.032
ASP	269	0.045	+/-	0.012
SER	270	0.037	+/-	0.013
ILE	271	0.064	+/-	0.020
SER	272	-0.010	+/-	0.019
SER	273	0.017	+/-	0.009
LYS	274	0.019	+/-	0.037
LEU	275	-0.016	+/-	0.043
LYS	276	-0.046	+/-	0.013
GLU	277	0.097	+/-	0.019
CYX	278	0.096	+/-	0.033
CYX	279	-0.001	+/-	0.020
GLU	280	0.066	+/-	0.014
LYS	281	0.023	+/-	0.028
PRO	282	-0.056	+/-	0.027
LEU	283	-0.074	+/-	0.085
LEU	284	-0.065	+/-	0.045
GLU	285	0.109	+/-	0.038
LYS	286	-0.256	+/-	0.309
SER	287	-2.337	+/-	0.915
HIE	288	0.107	+/-	0.119
CYX	289	0.005	+/-	0.120
ILE	290	-1.955	+/-	0.499
ALA	291	-1.320	+/-	0.768
GLU	292	0.434	+/-	0.194
VAL	293	-0.001	+/-	0.132
GLU	294	0.141	+/-	0.044
ASN	295	0.064	+/-	0.049
ASP	296	0.089	+/-	0.016

GLU	297	0.067	+/-	0.012
MET	298	0.006	+/-	0.004
PRO	299	-0.009	+/-	0.003
ALA	300	0.001	+/-	0.001
ASP	301	0.018	+/-	0.002
LEU	302	0.002	+/-	0.002
PRO	303	0.001	+/-	0.001
SER	304	0.002	+/-	0.001
LEU	305	0.003	+/-	0.001
ALA	306	-0.001	+/-	0.001
ALA	307	-0.001	+/-	0.001
ASP	308	0.020	+/-	0.003
PHE	309	-0.003	+/-	0.001
VAL	310	-0.000	+/-	0.001
GLU	311	0.009	+/-	0.001
SER	312	-0.000	+/-	0.001
LYS	313	-0.006	+/-	0.001
ASP	314	0.010	+/-	0.002
VAL	315	0.002	+/-	0.001
CYX	316	0.000	+/-	0.001
LYS	317	-0.008	+/-	0.001
ASN	318	0.002	+/-	0.001
TYR	319	-0.000	+/-	0.001
ALA	320	-0.000	+/-	0.001
GLU	321	0.014	+/-	0.002
ALA	322	0.001	+/-	0.001
LYS	323	-0.015	+/-	0.003
ASP	324	0.051	+/-	0.008
VAL	325	0.007	+/-	0.003
PHE	326	0.005	+/-	0.003
LEU	327	0.012	+/-	0.004
GLY	328	0.010	+/-	0.004
MET	329	0.006	+/-	0.005
PHE	330	0.011	+/-	0.005
LEU	331	0.045	+/-	0.013
TYR	332	-0.007	+/-	0.007
GLU	333	0.034	+/-	0.007
TYR	334	0.014	+/-	0.007
ALA	335	0.051	+/-	0.013
ARG	336	-0.051	+/-	0.012
ARG	337	-0.024	+/-	0.007
HIE	338	0.031	+/-	0.010
PRO	339	0.008	+/-	0.013
ASP	340	0.057	+/-	0.017
TYR	341	0.035	+/-	0.014
SER	342	0.038	+/-	0.015
VAL	343	0.060	+/-	0.039
VAL	344	0.003	+/-	0.013
LEU	345	0.026	+/-	0.014
LEU	346	0.037	+/-	0.012
LEU	347	0.017	+/-	0.011
ARG	348	-0.030	+/-	0.006
LEU	349	0.010	+/-	0.004
ALA	350	0.002	+/-	0.003
LYS	351	-0.030	+/-	0.003
THR	352	0.002	+/-	0.002
TYR	353	-0.001	+/-	0.001
GLU	354	0.031	+/-	0.004
THR	355	-0.002	+/-	0.001
THR	356	0.000	+/-	0.001

LEU	357	0.000	+/-	0.001
GLU	358	0.014	+/-	0.002
LYS	359	-0.010	+/-	0.002
CYX	360	-0.000	+/-	0.001
CYX	361	-0.001	+/-	0.001
ALA	362	-0.000	+/-	0.001
ALA	363	0.000	+/-	0.001
ALA	364	0.000	+/-	0.001
ASP	365	0.005	+/-	0.001
PRO	366	0.000	+/-	0.001
HIE	367	0.000	+/-	0.001
GLU	368	0.005	+/-	0.001
CYX	369	0.001	+/-	0.001
TYR	370	0.000	+/-	0.001
ALA	371	0.000	+/-	0.001
LYS	372	-0.007	+/-	0.001
VAL	373	0.002	+/-	0.001
PHE	374	-0.001	+/-	0.001
ASP	375	0.012	+/-	0.002
GLU	376	0.014	+/-	0.002
PHE	377	-0.002	+/-	0.001
LYS	378	-0.014	+/-	0.004
PRO	379	0.000	+/-	0.001
LEU	380	0.006	+/-	0.003
VAL	381	0.005	+/-	0.003
GLU	382	0.021	+/-	0.004
GLU	383	0.030	+/-	0.006
PRO	384	-0.003	+/-	0.003
GLN	385	0.002	+/-	0.003
ASN	386	0.000	+/-	0.001
LEU	387	0.005	+/-	0.003
ILE	388	0.002	+/-	0.003
LYS	389	-0.024	+/-	0.005
GLN	390	0.000	+/-	0.001
ASN	391	0.003	+/-	0.002
CYX	392	-0.001	+/-	0.001
GLU	393	0.014	+/-	0.002
LEU	394	0.000	+/-	0.001
PHE	395	-0.002	+/-	0.001
GLU	396	0.015	+/-	0.003
GLN	397	-0.001	+/-	0.001
LEU	398	0.000	+/-	0.001
GLY	399	-0.000	+/-	0.001
GLU	400	0.025	+/-	0.006
TYR	401	0.000	+/-	0.001
LYS	402	-0.007	+/-	0.001
PHE	403	-0.001	+/-	0.001
GLN	404	0.002	+/-	0.002
ASN	405	0.000	+/-	0.001
ALA	406	0.000	+/-	0.001
LEU	407	0.002	+/-	0.001
LEU	408	0.001	+/-	0.001
VAL	409	0.000	+/-	0.001
ARG	410	-0.013	+/-	0.002
TYR	411	-0.002	+/-	0.001
THR	412	0.000	+/-	0.001
LYS	413	-0.007	+/-	0.001
LYS	414	-0.013	+/-	0.002
VAL	415	0.002	+/-	0.001
PRO	416	-0.001	+/-	0.001

GLN	417	-0.000	+/-	0.001
VAL	418	0.001	+/-	0.001
SER	419	0.000	+/-	0.001
THR	420	0.002	+/-	0.001
PRO	421	0.001	+/-	0.001
THR	422	0.005	+/-	0.002
LEU	423	0.003	+/-	0.001
VAL	424	0.005	+/-	0.002
GLU	425	0.029	+/-	0.009
VAL	426	0.008	+/-	0.003
SER	427	0.002	+/-	0.002
ARG	428	-0.005	+/-	0.012
ASN	429	0.034	+/-	0.010
LEU	430	0.004	+/-	0.004
GLY	431	0.002	+/-	0.002
LYS	432	-0.011	+/-	0.013
VAL	433	0.011	+/-	0.007
GLY	434	0.002	+/-	0.002
SER	435	0.003	+/-	0.003
LYS	436	-0.026	+/-	0.017
CYX	437	0.006	+/-	0.007
CYX	438	-0.004	+/-	0.003
LYS	439	-0.034	+/-	0.008
HIE	440	0.006	+/-	0.007
PRO	441	-0.009	+/-	0.009
GLU	442	0.057	+/-	0.015
ALA	443	0.035	+/-	0.017
LYS	444	-0.020	+/-	0.068
ARG	445	-0.023	+/-	0.011
MET	446	0.060	+/-	0.027
PRO	447	0.022	+/-	0.024
CYX	448	0.063	+/-	0.024
ALA	449	0.004	+/-	0.011
GLU	450	0.129	+/-	0.037
ASP	451	0.332	+/-	0.154
TYR	452	-0.027	+/-	0.018
LEU	453	0.018	+/-	0.013
SER	454	0.034	+/-	0.025
VAL	455	0.027	+/-	0.015
VAL	456	0.020	+/-	0.010
LEU	457	0.023	+/-	0.011
ASN	458	0.049	+/-	0.018
GLN	459	0.020	+/-	0.009
LEU	460	0.009	+/-	0.005
CYX	461	0.005	+/-	0.005
VAL	462	0.005	+/-	0.005
LEU	463	-0.001	+/-	0.002
HIE	464	0.001	+/-	0.004
GLU	465	0.042	+/-	0.012
LYS	466	-0.020	+/-	0.004
THR	467	0.001	+/-	0.001
PRO	468	-0.001	+/-	0.001
VAL	469	-0.000	+/-	0.001
SER	470	0.001	+/-	0.001
ASP	471	0.011	+/-	0.002
ARG	472	-0.008	+/-	0.001
VAL	473	0.005	+/-	0.002
THR	474	0.002	+/-	0.001
LYS	475	-0.011	+/-	0.002
CYX	476	0.012	+/-	0.004

CYX	477	0.004	+/-	0.004
THR	478	0.001	+/-	0.003
GLU	479	0.036	+/-	0.006
SER	480	0.011	+/-	0.005
LEU	481	0.036	+/-	0.012
VAL	482	0.003	+/-	0.006
ASN	483	0.007	+/-	0.004
ARG	484	-0.007	+/-	0.017
ARG	485	-0.025	+/-	0.011
PRO	486	-0.010	+/-	0.003
CYX	487	0.005	+/-	0.003
PHE	488	-0.007	+/-	0.004
SER	489	-0.003	+/-	0.002
ALA	490	-0.001	+/-	0.001
LEU	491	0.001	+/-	0.001
GLU	492	0.011	+/-	0.002
VAL	493	0.000	+/-	0.001
ASP	494	0.007	+/-	0.001
GLU	495	0.006	+/-	0.001
THR	496	-0.000	+/-	0.001
TYR	497	-0.000	+/-	0.001
VAL	498	0.000	+/-	0.001
PRO	499	-0.000	+/-	0.001
LYS	500	-0.004	+/-	0.001
GLU	501	0.003	+/-	0.001
PHE	502	-0.000	+/-	0.001
ASN	503	-0.000	+/-	0.001
ALA	504	0.000	+/-	0.001
GLU	505	0.005	+/-	0.001
THR	506	0.000	+/-	0.001
PHE	507	-0.000	+/-	0.001
THR	508	0.000	+/-	0.001
PHE	509	-0.000	+/-	0.001
HIE	510	0.000	+/-	0.001
ALA	511	0.000	+/-	0.001
ASP	512	0.005	+/-	0.001
ILE	513	0.000	+/-	0.001
CYX	514	-0.000	+/-	0.001
THR	515	0.000	+/-	0.001
LEU	516	-0.000	+/-	0.001
SER	517	0.000	+/-	0.001
GLU	518	0.007	+/-	0.002
LYS	519	-0.016	+/-	0.004
GLU	520	0.014	+/-	0.003
ARG	521	-0.007	+/-	0.001
GLN	522	0.001	+/-	0.001
ILE	523	-0.000	+/-	0.001
LYS	524	-0.008	+/-	0.002
LYS	525	-0.007	+/-	0.001
GLN	526	0.001	+/-	0.001
THR	527	0.000	+/-	0.001
ALA	528	-0.000	+/-	0.001
LEU	529	0.000	+/-	0.001
VAL	530	0.001	+/-	0.001
GLU	531	0.006	+/-	0.001
LEU	532	-0.000	+/-	0.001
VAL	533	0.000	+/-	0.001
LYS	534	-0.009	+/-	0.001
HIE	535	-0.000	+/-	0.001
LYS	536	-0.003	+/-	0.001

PRO	537	0.000	+/-	0.001
LYS	538	-0.003	+/-	0.001
ALA	539	0.000	+/-	0.001
THR	540	-0.000	+/-	0.001
LYS	541	-0.007	+/-	0.002
GLU	542	0.005	+/-	0.001
GLN	543	0.000	+/-	0.001
LEU	544	-0.000	+/-	0.001
LYS	545	-0.005	+/-	0.001
ALA	546	-0.000	+/-	0.001
VAL	547	-0.000	+/-	0.001
MET	548	-0.000	+/-	0.001
ASP	549	0.004	+/-	0.001
ASP	550	0.002	+/-	0.001
PHE	551	-0.000	+/-	0.001
ALA	552	-0.000	+/-	0.001
ALA	553	-0.000	+/-	0.001
PHE	554	-0.000	+/-	0.001
VAL	555	-0.000	+/-	0.001
GLU	556	0.003	+/-	0.001
LYS	557	-0.002	+/-	0.001
CYX	558	-0.000	+/-	0.001
CYX	559	-0.000	+/-	0.001
LYS	560	-0.002	+/-	0.001
ALA	561	0.000	+/-	0.001
ASP	562	0.001	+/-	0.001
ASP	563	0.001	+/-	0.001
LYS	564	-0.001	+/-	0.001
GLU	565	0.002	+/-	0.001
THR	566	0.000	+/-	0.001
CYX	567	0.000	+/-	0.001
PHE	568	0.000	+/-	0.001
ALA	569	0.000	+/-	0.001
GLU	570	0.002	+/-	0.001
GLU	571	0.001	+/-	0.001
GLY	572	-0.000	+/-	0.001
LYS	573	-0.003	+/-	0.001
LYS	574	-0.001	+/-	0.001
LEU	575	-0.000	+/-	0.001
VAL	576	-0.000	+/-	0.001
ALA	577	-0.000	+/-	0.001
ALA	578	-0.000	+/-	0.001
SER	579	-0.000	+/-	0.001
GLN	580	-0.000	+/-	0.001
ALA	581	-0.000	+/-	0.001
ALA	582	-0.000	+/-	0.001
LEU	583	-0.000	+/-	0.001
GLY	584	0.000	+/-	0.001
LEU	585	0.002	+/-	0.001

Table S3. Ce6-HSA interactions in the heme binding pocket. ΔEbinding decomposed per residue. Ce6-HSA interactions in the Heme site. $\Delta E_{binding}$ decomposed per residue. Energetic value of the interactions and Std Dev.

ASP	1	0.003	+/-	0.001
ALA	2	0.001	+/-	0.001
HIE	3	0.000	+/-	0.001
LYS	4	-0.004	+/-	0.001
SER	5	0.001	+/-	0.001
GLU	6	0.018	+/-	0.002
VAL	7	0.006	+/-	0.002
ALA	8	0.000	+/-	0.001
HIE	9	0.003	+/-	0.001
ARG	10	-0.008	+/-	0.002
PHE	11	-0.001	+/-	0.002
LYS	12	-0.011	+/-	0.002
ASP	13	0.018	+/-	0.003
LEU	14	0.013	+/-	0.003
GLY	15	-0.004	+/-	0.004
GLU	16	0.029	+/-	0.005
GLU	17	0.047	+/-	0.024
ASN	18	0.021	+/-	0.010
PHE	19	-0.007	+/-	0.009
LYS	20	-0.004	+/-	0.013
ALA	21	-0.024	+/-	0.016
LEU	22	0.012	+/-	0.013
VAL	23	0.011	+/-	0.012
LEU	24	-0.083	+/-	0.036
ILE	25	-0.047	+/-	0.021
ALA	26	0.024	+/-	0.010
PHE	27	-0.020	+/-	0.011
ALA	28	0.008	+/-	0.014
GLN	29	0.082	+/-	0.016
TYR	30	0.010	+/-	0.006
LEU	31	0.022	+/-	0.007
GLN	32	0.062	+/-	0.016
GLN	33	0.023	+/-	0.011
CYS	34	0.018	+/-	0.007
PRO	35	-0.002	+/-	0.007
PHE	36	-0.047	+/-	0.011
GLU	37	0.081	+/-	0.026
ASP	38	0.062	+/-	0.007
HIE	39	-0.014	+/-	0.011
VAL	40	0.013	+/-	0.006
LYS	41	-0.024	+/-	0.006
LEU	42	0.023	+/-	0.005
VAL	43	0.017	+/-	0.005
ASN	44	0.002	+/-	0.003
GLU	45	0.021	+/-	0.003
VAL	46	0.017	+/-	0.004
THR	47	0.001	+/-	0.004
GLU	48	0.012	+/-	0.002
PHE	49	0.002	+/-	0.001
ALA	50	0.001	+/-	0.001
LYS	51	-0.019	+/-	0.002
THR	52	-0.000	+/-	0.001
CYX	53	-0.000	+/-	0.001
VAL	54	-0.001	+/-	0.001
ALA	55	-0.000	+/-	0.001

ASP	56	0.005	+/-	0.001
GLU	57	0.005	+/-	0.001
SER	58	-0.000	+/-	0.001
ALA	59	0.000	+/-	0.001
GLU	60	0.006	+/-	0.001
ASN	61	0.001	+/-	0.001
CYX	62	0.001	+/-	0.001
ASP	63	0.007	+/-	0.001
LYS	64	-0.008	+/-	0.001
SER	65	0.004	+/-	0.001
LEU	66	0.015	+/-	0.004
HIE	67	-0.004	+/-	0.003
THR	68	0.003	+/-	0.002
LEU	69	0.012	+/-	0.004
PHE	70	-0.008	+/-	0.003
GLY	71	-0.001	+/-	0.002
ASP	72	0.012	+/-	0.002
LYS	73	-0.010	+/-	0.002
LEU	74	0.012	+/-	0.004
CYX	75	-0.004	+/-	0.002
THR	76	0.000	+/-	0.001
VAL	77	0.004	+/-	0.002
ALA	78	-0.001	+/-	0.001
THR	79	0.003	+/-	0.003
LEU	80	0.006	+/-	0.002
ARG	81	-0.014	+/-	0.002
GLU	82	0.023	+/-	0.004
THR	83	0.009	+/-	0.003
TYR	84	-0.019	+/-	0.006
GLY	85	0.000	+/-	0.001
GLU	86	0.016	+/-	0.003
MET	87	0.001	+/-	0.002
ALA	88	-0.002	+/-	0.001
ASP	89	0.011	+/-	0.001
CYX	90	0.001	+/-	0.001
CYX	91	-0.001	+/-	0.001
ALA	92	-0.001	+/-	0.001
LYS	93	-0.009	+/-	0.001
GLN	94	0.001	+/-	0.001
GLU	95	0.010	+/-	0.001
PRO	96	0.000	+/-	0.001
GLU	97	0.010	+/-	0.001
ARG	98	-0.010	+/-	0.002
ASN	99	0.007	+/-	0.003
GLU	100	0.017	+/-	0.003
CYX	101	0.007	+/-	0.002
PHE	102	0.003	+/-	0.002
LEU	103	0.004	+/-	0.002
GLN	104	0.000	+/-	0.002
HIE	105	0.003	+/-	0.003
LYS	106	0.016	+/-	0.021
ASP	107	0.063	+/-	0.011
ASP	108	0.066	+/-	0.014
ASN	109	0.052	+/-	0.015
PRO	110	0.023	+/-	0.015
ASN	111	0.044	+/-	0.024
LEU	112	0.085	+/-	0.030
PRO	113	0.009	+/-	0.048
ARG	114	-3.897	+/-	3.616
LEU	115	-0.304	+/-	0.844

VAL	116	-0.087	+/-	0.308
ARG	117	-8.908	+/-	1.240
PRO	118	-0.779	+/-	0.232
GLU	119	0.114	+/-	0.044
VAL	120	-0.042	+/-	0.031
ASP	121	0.060	+/-	0.016
VAL	122	0.008	+/-	0.034
MET	123	-0.937	+/-	0.234
CYX	124	-0.017	+/-	0.013
THR	125	-0.006	+/-	0.011
ALA	126	-0.088	+/-	0.033
PHE	127	-0.059	+/-	0.026
HIE	128	0.035	+/-	0.010
ASP	129	0.089	+/-	0.010
ASN	130	-0.015	+/-	0.021
GLU	131	0.057	+/-	0.039
GLU	132	0.056	+/-	0.040
THR	133	-0.064	+/-	0.035
PHE	134	-0.442	+/-	0.228
LEU	135	-0.576	+/-	0.336
LYS	136	-0.012	+/-	0.076
LYS	137	-0.594	+/-	0.236
TYR	138	-4.376	+/-	0.902
LEU	139	-0.920	+/-	0.274
TYR	140	-0.257	+/-	0.071
GLU	141	-0.398	+/-	0.267
ILE	142	-3.013	+/-	0.504
ALA	143	-0.047	+/-	0.048
ARG	144	0.216	+/-	0.072
ARG	145	0.180	+/-	0.250
HIE	146	-0.038	+/-	0.079
PRO	147	-0.004	+/-	0.015
TYR	148	0.003	+/-	0.009
PHE	149	-0.021	+/-	0.008
TYR	150	-0.039	+/-	0.015
ALA	151	-0.061	+/-	0.020
PRO	152	-0.005	+/-	0.018
GLU	153	0.054	+/-	0.049
LEU	154	-0.974	+/-	0.242
LEU	155	-0.152	+/-	0.063
PHE	156	-0.192	+/-	0.069
PHE	157	-1.069	+/-	0.333
ALA	158	-1.224	+/-	0.402
LYS	159	0.074	+/-	0.081
ARG	160	-0.436	+/-	0.126
TYR	161	-3.648	+/-	0.714
LYS	162	-0.295	+/-	0.274
ALA	163	-0.019	+/-	0.031
ALA	164	-0.195	+/-	0.062
PHE	165	-1.004	+/-	0.296
THR	166	0.046	+/-	0.016
GLU	167	0.087	+/-	0.013
CYX	168	-0.023	+/-	0.016
CYX	169	0.017	+/-	0.008
GLN	170	0.021	+/-	0.007
ALA	171	0.009	+/-	0.005
ALA	172	0.008	+/-	0.003
ASP	173	0.072	+/-	0.015
LYS	174	-0.006	+/-	0.014
ALA	175	-0.009	+/-	0.011

ALA	176	0.007	+/-	0.005
CYX	177	0.018	+/-	0.019
LEU	178	-0.184	+/-	0.083
LEU	179	-0.213	+/-	0.173
PRO	180	0.020	+/-	0.028
LYS	181	-0.039	+/-	0.039
LEU	182	-1.108	+/-	0.285
ASP	183	0.081	+/-	0.113
GLU	184	0.053	+/-	0.039
LEU	185	-0.469	+/-	0.109
ARG	186	-5.390	+/-	2.170
ASP	187	-0.001	+/-	0.111
GLU	188	-0.014	+/-	0.051
GLY	189	-0.252	+/-	0.266
LYS	190	-0.323	+/-	0.571
ALA	191	-0.015	+/-	0.017
SER	192	-0.001	+/-	0.053
SER	193	0.012	+/-	0.079
ALA	194	0.014	+/-	0.009
LYS	195	0.055	+/-	0.032
GLN	196	0.069	+/-	0.023
ARG	197	-0.012	+/-	0.018
LEU	198	0.016	+/-	0.006
LYS	199	0.020	+/-	0.015
CYX	200	0.000	+/-	0.004
ALA	201	0.002	+/-	0.003
SER	202	-0.000	+/-	0.002
LEU	203	0.001	+/-	0.002
GLN	204	0.002	+/-	0.003
LYS	205	-0.022	+/-	0.003
PHE	206	-0.004	+/-	0.003
GLY	207	0.001	+/-	0.001
GLU	208	0.010	+/-	0.002
ARG	209	-0.007	+/-	0.001
ALA	210	0.002	+/-	0.001
PHE	211	-0.002	+/-	0.002
LYS	212	-0.010	+/-	0.002
ALA	213	0.002	+/-	0.001
TRP	214	-0.004	+/-	0.002
ALA	215	0.001	+/-	0.001
VAL	216	0.001	+/-	0.001
ALA	217	0.002	+/-	0.001
ARG	218	-0.017	+/-	0.004
LEU	219	0.001	+/-	0.001
SER	220	-0.000	+/-	0.001
GLN	221	0.002	+/-	0.001
ARG	222	-0.017	+/-	0.003
PHE	223	-0.002	+/-	0.001
PRO	224	-0.002	+/-	0.001
LYS	225	-0.006	+/-	0.001
ALA	226	-0.000	+/-	0.001
GLU	227	0.004	+/-	0.001
PHE	228	0.000	+/-	0.001
ALA	229	0.000	+/-	0.001
GLU	230	0.006	+/-	0.001
VAL	231	0.001	+/-	0.001
SER	232	0.000	+/-	0.001
LYS	233	-0.005	+/-	0.001
LEU	234	0.003	+/-	0.001
VAL	235	0.002	+/-	0.001

THR	236	0.001	+/-	0.001
ASP	237	0.012	+/-	0.001
LEU	238	0.009	+/-	0.003
THR	239	0.001	+/-	0.002
LYS	240	-0.007	+/-	0.002
VAL	241	0.013	+/-	0.004
HIE	242	0.002	+/-	0.003
THR	243	0.003	+/-	0.002
GLU	244	0.024	+/-	0.004
CYX	245	0.018	+/-	0.004
CYX	246	0.003	+/-	0.004
HIE	247	0.009	+/-	0.003
GLY	248	0.010	+/-	0.004
ASP	249	0.022	+/-	0.004
LEU	250	0.019	+/-	0.006
LEU	251	0.005	+/-	0.003
GLU	252	0.017	+/-	0.003
CYX	253	0.024	+/-	0.006
ALA	254	0.008	+/-	0.004
ASP	255	0.013	+/-	0.002
ASP	256	0.016	+/-	0.002
ARG	257	- 0.003	+/-	0.010
ALA	258	-0.001	+/-	0.002
ASP	259	0.009	+/-	0.002
LEU	260	0.007	+/-	0.003
ALA	261	0.001	+/-	0.002
LYS	262	-0.013	+/-	0.002
TYR	263	0.000	+/-	0.001
ILE	264	0.002	+/-	0.001
CYX	265	-0.002	+/-	0.001
GLU	266	0.006	+/-	0.001
ASN	267	-0.001	+/-	0.001
GLN	268	0.000	+/-	0.001
ASP	269	0.005	+/-	0.001
SER	270	-0.000	+/-	0.001
ILE	271	0.000	+/-	0.001
SER	272	-0.000	+/-	0.001
SER	273	0.000	+/-	0.001
LYS	274	-0.010	+/-	0.002
LEU	275	0.003	+/-	0.001
LYS	276	-0.007	+/-	0.001
GLU	277	0.022	+/-	0.006
CYX	278	0.008	+/-	0.003
CYX	279	-0.002	+/-	0.001
GLU	280	0.013	+/-	0.004
LYS	281	-0.012	+/-	0.005
PRO	282	-0.008	+/-	0.005
LEU	283	0.020	+/-	0.005
LEU	284	0.018	+/-	0.007
GLU	285	0.048	+/-	0.016
LYS	286	0.000	+/-	0.005
SER	287	0.014	+/-	0.006
HIE	288	0.011	+/-	0.010
CYX	289	0.009	+/-	0.004
ILE	290	0.004	+/-	0.003
ALA	291	0.004	+/-	0.004
GLU	292	0.053	+/-	0.013
VAL	293	0.004	+/-	0.002
GLU	294	0.023	+/-	0.008
ASN	295	0.001	+/-	0.001

ASP	296	0.008	+/-	0.001
GLU	297	0.008	+/-	0.001
MET	298	-0.000	+/-	0.001
PRO	299	0.000	+/-	0.001
ALA	300	-0.000	+/-	0.001
ASP	301	0.002	+/-	0.001
LEU	302	-0.000	+/-	0.001
PRO	303	0.000	+/-	0.001
SER	304	-0.000	+/-	0.001
LEU	305	-0.000	+/-	0.001
ALA	306	-0.000	+/-	0.001
ALA	307	-0.000	+/-	0.001
ASP	308	0.002	+/-	0.001
PHE	309	-0.000	+/-	0.001
VAL	310	-0.000	+/-	0.001
GLU	311	0.001	+/-	0.001
SER	312	-0.000	+/-	0.001
LYS	313	-0.001	+/-	0.001
ASP	314	0.001	+/-	0.001
VAL	315	0.000	+/-	0.001
CYX	316	0.000	+/-	0.001
LYS	317	-0.001	+/-	0.001
ASN	318	0.000	+/-	0.001
TYR	319	0.000	+/-	0.001
ALA	320	0.000	+/-	0.001
GLU	321	0.002	+/-	0.001
ALA	322	0.000	+/-	0.001
LYS	323	-0.003	+/-	0.001
ASP	324	0.005	+/-	0.001
VAL	325	0.000	+/-	0.001
PHE	326	0.000	+/-	0.001
LEU	327	0.000	+/-	0.001
GLY	328	0.000	+/-	0.001
MET	329	0.000	+/-	0.001
PHE	330	0.000	+/-	0.001
LEU	331	0.001	+/-	0.001
TYR	332	0.000	+/-	0.001
GLU	333	0.004	+/-	0.001
TYR	334	0.000	+/-	0.001
ALA	335	0.001	+/-	0.001
ARG	336	-0.005	+/-	0.001
ARG	337	-0.004	+/-	0.001
HIE	338	0.001	+/-	0.001
PRO	339	0.000	+/-	0.001
ASP	340	0.013	+/-	0.003
TYR	341	0.002	+/-	0.001
SER	342	0.001	+/-	0.001
VAL	343	0.005	+/-	0.002
VAL	344	0.000	+/-	0.001
LEU	345	0.001	+/-	0.001
LEU	346	0.001	+/-	0.001
LEU	347	0.000	+/-	0.001
ARG	348	-0.011	+/-	0.001
LEU	349	-0.000	+/-	0.001
ALA	350	-0.000	+/-	0.001
LYS	351	-0.007	+/-	0.001
THR	352	-0.000	+/-	0.001
TYR	353	-0.000	+/-	0.001
GLU	354	0.005	+/-	0.001
THR	355	-0.000	+/-	0.001

THR	356	-0.000	+/-	0.001
LEU	357	-0.000	+/-	0.001
GLU	358	0.003	+/-	0.001
LYS	359	-0.002	+/-	0.001
CYX	360	-0.000	+/-	0.001
CYX	361	-0.000	+/-	0.001
ALA	362	-0.000	+/-	0.001
ALA	363	0.000	+/-	0.001
ALA	364	-0.000	+/-	0.001
ASP	365	0.001	+/-	0.001
PRO	366	0.000	+/-	0.001
HIE	367	0.000	+/-	0.001
GLU	368	0.001	+/-	0.001
CYX	369	0.000	+/-	0.001
TYR	370	0.000	+/-	0.001
ALA	371	0.000	+/-	0.001
LYS	372	-0.002	+/-	0.001
VAL	373	0.000	+/-	0.001
PHE	374	0.000	+/-	0.001
ASP	375	0.003	+/-	0.001
GLU	376	0.003	+/-	0.001
PHE	377	0.000	+/-	0.001
LYS	378	-0.003	+/-	0.001
PRO	379	0.000	+/-	0.001
LEU	380	0.001	+/-	0.001
VAL	381	0.001	+/-	0.001
GLU	382	0.007	+/-	0.001
GLU	383	0.014	+/-	0.002
PRO	384	-0.000	+/-	0.001
GLN	385	0.001	+/-	0.001
ASN	386	0.001	+/-	0.001
LEU	387	0.003	+/-	0.001
ILE	388	0.001	+/-	0.001
LYS	389	-0.009	+/-	0.002
GLN	390	0.001	+/-	0.001
ASN	391	0.003	+/-	0.002
CYX	392	-0.000	+/-	0.001
GLU	393	0.010	+/-	0.002
LEU	394	0.001	+/-	0.001
PHE	395	-0.001	+/-	0.001
GLU	396	0.013	+/-	0.004
GLN	397	-0.000	+/-	0.001
LEU	398	0.000	+/-	0.001
GLY	399	0.000	+/-	0.001
GLU	400	0.061	+/-	0.023
TYR	401	-0.001	+/-	0.004
LYS	402	-0.018	+/-	0.004
PHE	403	0.000	+/-	0.002
GLN	404	0.017	+/-	0.008
ASN	405	0.002	+/-	0.002
ALA	406	0.000	+/-	0.001
LEU	407	0.005	+/-	0.003
LEU	408	0.005	+/-	0.003
VAL	409	-0.000	+/-	0.001
ARG	410	-0.015	+/-	0.002
TYR	411	-0.002	+/-	0.001
THR	412	-0.000	+/-	0.001
LYS	413	-0.013	+/-	0.002
LYS	414	-0.012	+/-	0.002
VAL	415	0.003	+/-	0.001

PRO	416	-0.000	+/-	0.001
GLN	417	0.002	+/-	0.001
VAL	418	0.005	+/-	0.002
SER	419	0.004	+/-	0.003
THR	420	0.016	+/-	0.007
PRO	421	-0.000	+/-	0.004
THR	422	0.018	+/-	0.007
LEU	423	0.011	+/-	0.006
VAL	424	0.036	+/-	0.012
GLU	425	0.110	+/-	0.045
VAL	426	0.009	+/-	0.007
SER	427	0.009	+/-	0.006
ARG	428	0.049	+/-	0.064
ASN	429	0.041	+/-	0.014
LEU	430	0.004	+/-	0.004
GLY	431	0.007	+/-	0.005
LYS	432	0.047	+/-	0.034
VAL	433	0.008	+/-	0.005
GLY	434	-0.000	+/-	0.002
SER	435	0.003	+/-	0.005
LYS	436	-0.019	+/-	0.025
CYX	437	-0.003	+/-	0.002
CYX	438	-0.002	+/-	0.001
LYS	439	-0.023	+/-	0.005
HIE	440	-0.001	+/-	0.001
PRO	441	0.000	+/-	0.001
GLU	442	0.011	+/-	0.003
ALA	443	0.001	+/-	0.001
LYS	444	-0.013	+/-	0.003
ARG	445	-0.012	+/-	0.002
MET	446	0.005	+/-	0.002
PRO	447	0.002	+/-	0.002
CYX	448	0.019	+/-	0.005
ALA	449	0.001	+/-	0.003
GLU	450	0.035	+/-	0.007
ASP	451	0.060	+/-	0.012
TYR	452	-0.019	+/-	0.014
LEU	453	0.008	+/-	0.004
SER	454	0.026	+/-	0.010
VAL	455	0.026	+/-	0.009
VAL	456	0.013	+/-	0.007
LEU	457	0.013	+/-	0.005
ASN	458	0.039	+/-	0.013
GLN	459	0.049	+/-	0.016
LEU	460	0.005	+/-	0.003
CYX	461	0.003	+/-	0.004
VAL	462	0.014	+/-	0.007
LEU	463	0.004	+/-	0.004
HIE	464	-0.001	+/-	0.002
GLU	465	0.062	+/-	0.025
LYS	466	-0.044	+/-	0.011
THR	467	0.003	+/-	0.002
PRO	468	0.002	+/-	0.001
VAL	469	0.000	+/-	0.001
SER	470	0.000	+/-	0.001
ASP	471	0.012	+/-	0.002
ARG	472	-0.009	+/-	0.001
VAL	473	0.003	+/-	0.001
THR	474	0.000	+/-	0.001
LYS	475	-0.008	+/-	0.001

CYX	476	0.003	+/-	0.002
CYX	477	-0.000	+/-	0.001
THR	478	-0.001	+/-	0.001
GLU	479	0.012	+/-	0.001
SER	480	0.001	+/-	0.001
LEU	481	0.004	+/-	0.002
VAL	482	0.001	+/-	0.001
ASN	483	0.001	+/-	0.001
ARG	484	-0.014	+/-	0.004
ARG	485	-0.012	+/-	0.002
PRO	486	-0.004	+/-	0.001
CYX	487	0.003	+/-	0.001
PHE	488	-0.004	+/-	0.002
SER	489	-0.002	+/-	0.001
ALA	490	-0.000	+/-	0.001
LEU	491	0.001	+/-	0.001
GLU	492	0.011	+/-	0.001
VAL	493	-0.000	+/-	0.001
ASP	494	0.012	+/-	0.002
GLU	495	0.007	+/-	0.001
THR	496	0.000	+/-	0.001
TYR	497	-0.001	+/-	0.001
VAL	498	0.001	+/-	0.001
PRO	499	-0.001	+/-	0.001
LYS	500	-0.018	+/-	0.006
GLU	501	0.013	+/-	0.002
PHE	502	-0.001	+/-	0.001
ASN	503	0.001	+/-	0.002
ALA	504	0.002	+/-	0.001
GLU	505	0.036	+/-	0.009
THR	506	0.009	+/-	0.003
PHE	507	0.000	+/-	0.002
THR	508	0.006	+/-	0.003
PHE	509	-0.007	+/-	0.003
HIE	510	0.019	+/-	0.010
ALA	511	0.023	+/-	0.010
ASP	512	0.236	+/-	0.054
ILE	513	0.044	+/-	0.019
CYX	514	0.035	+/-	0.022
THR	515	-0.014	+/-	0.102
LEU	516	0.046	+/-	0.068
SER	517	-0.081	+/-	0.049
GLU	518	0.084	+/-	0.014
LYS	519	0.129	+/-	0.079
GLU	520	-0.017	+/-	0.421
ARG	521	-0.027	+/-	0.030
GLN	522	0.042	+/-	0.018
ILE	523	0.028	+/-	0.017
LYS	524	-0.075	+/-	0.034
LYS	525	-0.034	+/-	0.009
GLN	526	0.026	+/-	0.009
THR	527	0.004	+/-	0.006
ALA	528	-0.002	+/-	0.002
LEU	529	0.004	+/-	0.003
VAL	530	0.005	+/-	0.003
GLU	531	0.031	+/-	0.006
LEU	532	-0.000	+/-	0.001
VAL	533	0.000	+/-	0.001
LYS	534	-0.026	+/-	0.002
HIE	535	-0.001	+/-	0.001

LYS	536	-0.009	+/-	0.001
PRO	537	-0.000	+/-	0.001
LYS	538	-0.007	+/-	0.001
ALA	539	0.000	+/-	0.001
THR	540	0.001	+/-	0.001
LYS	541	-0.013	+/-	0.002
GLU	542	0.014	+/-	0.002
GLN	543	0.002	+/-	0.001
LEU	544	0.003	+/-	0.001
LYS	545	-0.024	+/-	0.004
ALA	546	0.001	+/-	0.001
VAL	547	0.004	+/-	0.001
MET	548	0.003	+/-	0.002
ASP	549	0.031	+/-	0.004
ASP	550	0.021	+/-	0.002
PHE	551	0.001	+/-	0.002
ALA	552	0.002	+/-	0.002
ALA	553	0.001	+/-	0.001
PHE	554	0.003	+/-	0.002
VAL	555	0.019	+/-	0.007
GLU	556	0.066	+/-	0.013
LYS	557	-0.022	+/-	0.003
CYX	558	0.006	+/-	0.003
CYX	559	0.001	+/-	0.003
LYS	560	-0.038	+/-	0.008
ALA	561	0.003	+/-	0.003
ASP	562	0.037	+/-	0.012
ASP	563	0.046	+/-	0.024
LYS	564	-0.024	+/-	0.004
GLU	565	0.051	+/-	0.016
THR	566	0.000	+/-	0.001
CYX	567	0.003	+/-	0.002
PHE	568	-0.007	+/-	0.003
ALA	569	-0.001	+/-	0.001
GLU	570	0.023	+/-	0.004
GLU	571	0.019	+/-	0.002
GLY	572	-0.001	+/-	0.001
LYS	573	-0.028	+/-	0.005
LYS	574	-0.017	+/-	0.002
LEU	575	-0.001	+/-	0.001
VAL	576	-0.000	+/-	0.001
ALA	577	-0.001	+/-	0.001
ALA	578	-0.001	+/-	0.001
SER	579	-0.001	+/-	0.001
GLN	580	-0.000	+/-	0.001
ALA	581	-0.000	+/-	0.001
ALA	582	-0.000	+/-	0.001
LEU	583	0.000	+/-	0.001
GLY	584	0.000	+/-	0.001
LEU	585	0.007	+/-	0.002

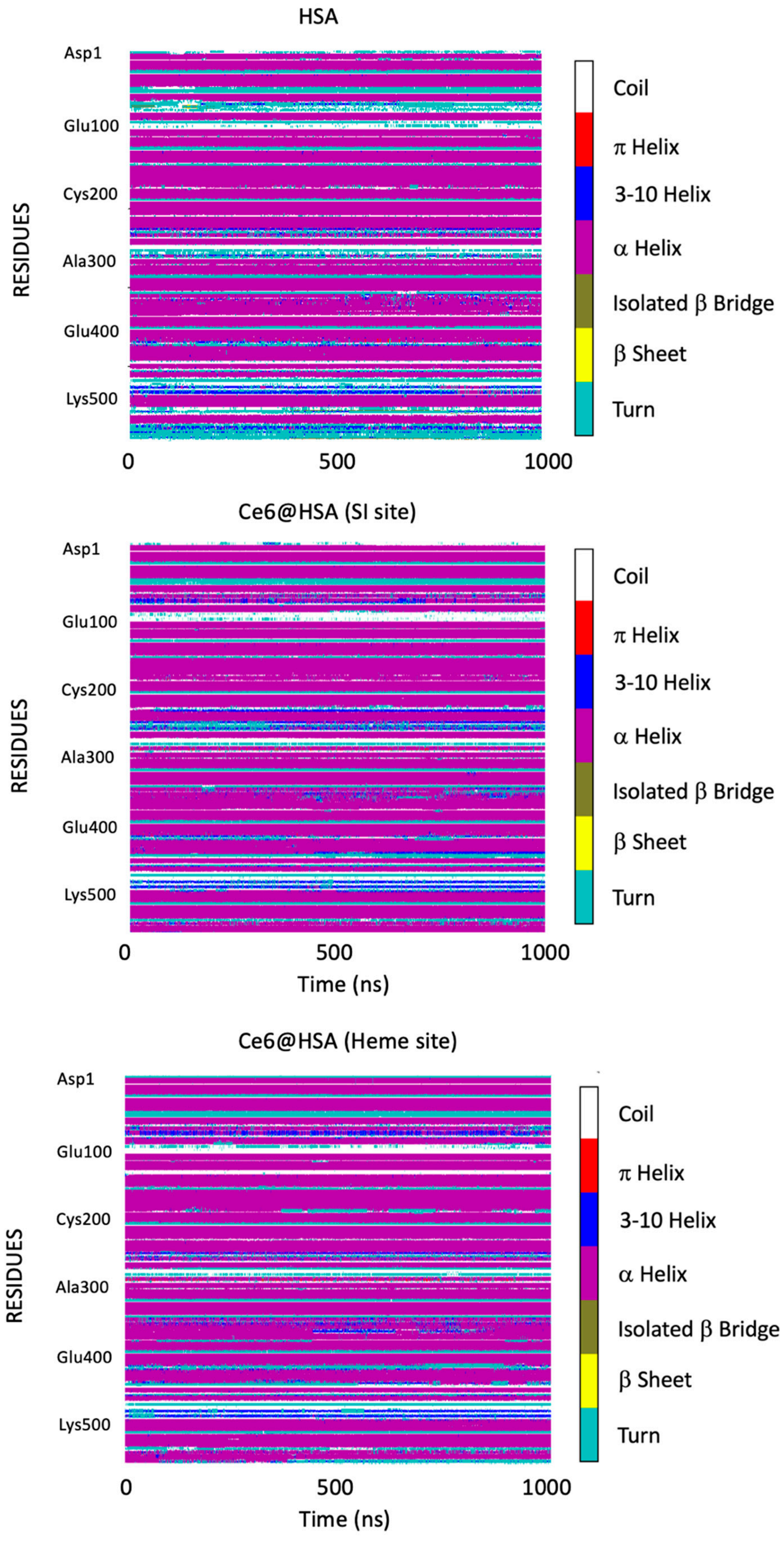

Figure S3. Secondary structure analysis of HSA, Ce6@HSA (SI site) and Ce6@HSA (Heme site) by using VMD timeline for 1 μs simulations.

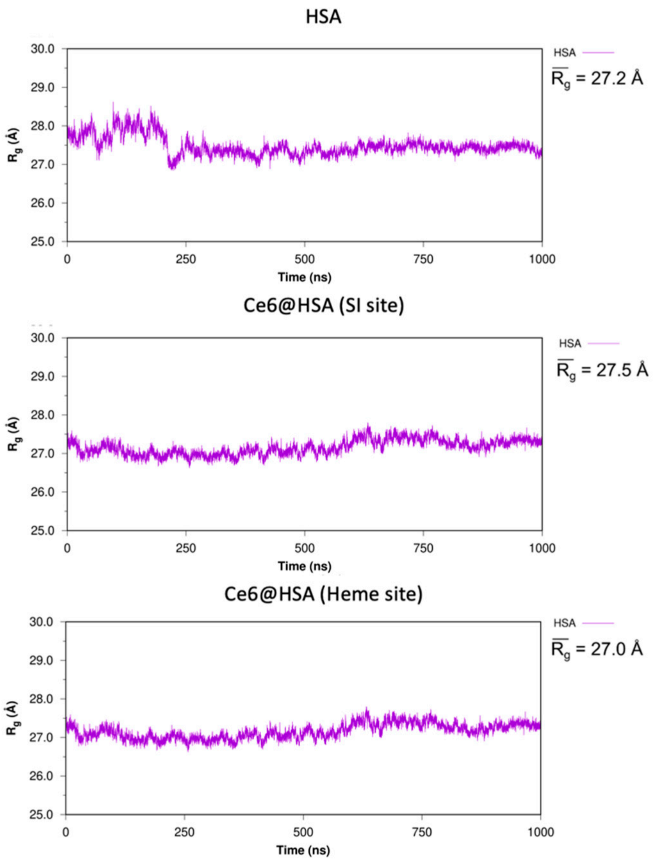

Figure S4. Radius of gyration of HSA, Ce6@HSA (SI site) and Ce6@HSA (Heme site) during 1 μs of MD simulations

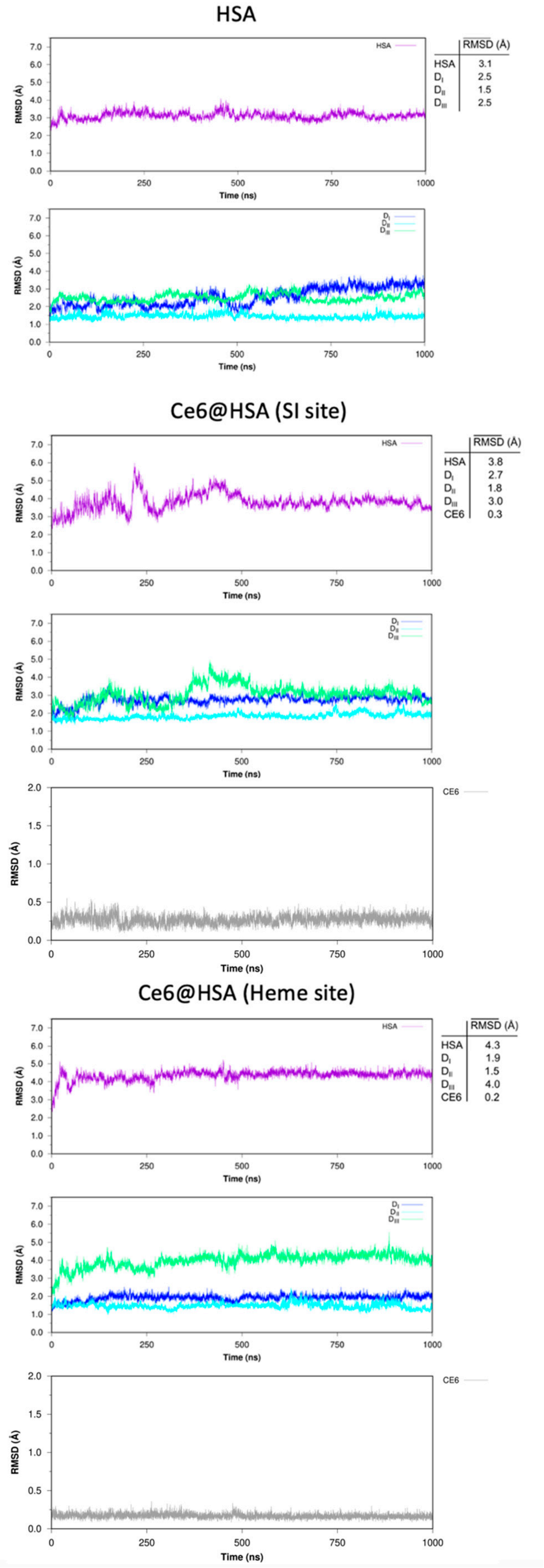

Figure S5. RMSD of the protein, of each protein domain and of Ce6 in HSA, Ce6@HSA (SI site) and Ce6@HSA (Heme site) during 1 µs of MD simulations
